# Decreases in Gap Junction Coupling Recovers Ca^2+^ and Insulin Secretion in Neonatal Diabetes Mellitus, Dependent on Beta Cell Heterogeneity and Noise

**DOI:** 10.1371/journal.pcbi.1005116

**Published:** 2016-09-28

**Authors:** Aleena M. Notary, Matthew J. Westacott, Thomas H. Hraha, Marina Pozzoli, Richard K. P. Benninger

**Affiliations:** 1 Department of Bioengineering, University of Colorado, Anschutz Medical campus, Aurora, Colorado, United States of America; 2 Barbara Davis Center for Diabetes, University of Colorado, Anschutz Medical campus, Aurora, Colorado, United States of America; University of Virginia, UNITED STATES

## Abstract

Diabetes is caused by dysfunction to β-cells in the islets of Langerhans, disrupting insulin secretion and glucose homeostasis. Gap junction-mediated electrical coupling between β-cells in the islet plays a major role in coordinating a pulsatile secretory response at elevated glucose and suppressing insulin secretion at basal glucose. Previously, we demonstrated that a critical number of inexcitable cells can rapidly suppress the overall islet response, as a result of gap junction coupling. This was demonstrated in a murine model of Neonatal Diabetes Mellitus (NDM) involving expression of ATP-insensitive K_ATP_ channels, and by a multi-cellular computational model of islet electrical activity. Here we examined the mechanisms by which gap junction coupling contributes to islet dysfunction in NDM. We first verified the computational model against [Ca^2+^] and insulin secretion measurements in islets expressing ATP-insensitive K_ATP_ channels under different levels of gap junction coupling. We then applied this model to predict how different K_ATP_ channel mutations found in NDM suppress [Ca^2+^], and the role of gap junction coupling in this suppression. We further extended the model to account for stochastic noise and insulin secretion dynamics. We found experimentally and in the islet model that reductions in gap junction coupling allow progressively greater glucose-stimulated [Ca^2+^] and insulin secretion following expression of ATP-insensitive K_ATP_ channels. The model demonstrated good correspondence between suppression of [Ca^2+^] and clinical presentation of different NDM mutations. Significant recoveries in [Ca^2+^] and insulin secretion were predicted for many mutations upon reductions in gap junction coupling, where stochastic noise played a significant role in the recoveries. These findings provide new understanding how the islet functions as a multicellular system and for the role of gap junction channels in exacerbating the effects of decreased cellular excitability. They further suggest novel therapeutic options for NDM and other monogenic forms of diabetes.

## Author Summary

Diabetes is a disease reaching a global epidemic, which results from dysfunction to the islets of Langerhans in the pancreas and their ability to secrete the hormone insulin to regulate glucose homeostasis. Islets are multicellular structures that show extensive coupling between heterogeneous cellular units; and central to the causes of diabetes is a dysfunction to these cellular units and their interactions. Understanding the inter-relationship between structure and function is challenging in biological systems, but is crucial to the cause of disease and discovering therapeutic targets. With the goal of further characterizing the islet of Langerhans and its excitable behavior, we examined the role of important channels in the islet where dysfunction is linked to or causes diabetes. Advances in our ability to computationally model perturbations in physiological systems has allowed for the testing of hypothesis quickly, in systems that are not experimentally accessible. Using an experimentally validated model and modeling human mutations, we discover that monogenic forms of diabetes may be remedied by a reduction in electrical coupling between cells; either alone or in conjunction with pharmacological intervention. Knowledge of biological systems in general is also helped by these findings, in that small changes to cellular elements may lead to major disruptions in the overall system. This may then be overcome by allowing the system components to function independently in the presence of dysfunction to individual cells.

## Introduction

Multi-cellular biological systems are composed of cellular elements with distinct characteristics, which function collectively as a result of dynamic interactions. While the function of a multicellular system is dependent on the characteristics of its constituent cells, understanding such systems is complicated by the action of cellular coupling and system architecture. Furthermore, cellular heterogeneity and noise complicate assessment of the function of individual cells. As a result, changes in the behavior of individual cells can often lead to unexpected changes in the system behavior. Many diseases, both acute and chronic, arise through genetic variations that impact molecular and cellular function. Given the complexities of multi-cellular systems, effectively predicting how molecular and cellular dysfunction lead to tissue and organ dysfunction and cause disease is challenging. One approach to describe dynamic multicellular systems is using network theory, which distinguishes network structure and cellular behavior to understand how distinct functions can emerge from coupled systems [1,2].

Islets of Langerhans located in the pancreas exhibit complex multicellular behavior. Islets are small (~1000 cells) micro-organs, where the primary cellular elements are insulin secreting β-cells. Death or dysfunction to β-cells and a reduction or absence of insulin secretion is the main cause of diabetes. β-cells are excitable, where glucose-stimulated insulin secretion is driven by electrical activity. The increased metabolism of glucose following blood glucose elevation increases ATP/ADP and inhibits ATP-sensitive K+ (K_ATP_) channels. The resulting membrane depolarization activates bursts of action potentials and elevates intracellular free-calcium activity ([Ca^2+^]) in the form of oscillations which triggers pulses of insulin granule exocytosis [3–5]. β-cells in the islet are electrically coupled by Connexin36 (Cx36) gap junction channels [6–8]. As a result of electrical coupling, [Ca^2+^] oscillations are coordinated under elevated glucose and uniformly silent under basal glucose [9–12].

Neonatal diabetes mellitus (NDM) is a monogenic form of diabetes that arises in the first 6 months of life. It can have a transient phenotype (TNDM); be permanent (PNDM) where the patient requires lifelong treatment with insulin or sulfonylureas; and may display neurological features (DEND) [13]. The majority of NDM cases have mutations on the *KCNJ11* and *ABCC8* genes [13–15], which encode the Kir6.2 and SUR1 subunits of the K_ATP_ channel. Mutations to these subunits often result in reduced sensitivity to ATP and increased open channel stability [14,16–22]; thereby limiting electrical activity and insulin secretion. This was shown in a murine model of NDM [23,24], where expression of ATP insensitive K_ATP_ channels led to blunted glucose-stimulated [Ca^2+^], insulin secretion and sharply elevated blood glucose levels. Sulfonylurea therapy to inhibit K_ATP_ channel is effective in many NDM patients with *KCNJ11* and *ABCC8* mutations [25], especially if treated early [26]. However some NDM patients with specific *KCNJ11* and *ABCC8* mutations do not respond to sulfonylureas and require life-long insulin therapy [25].

Our prior studies have shown that as a result of electrical coupling, small changes in β-cell excitability (e.g. upon glucose stimulation) leads to a rapid change in global islet behavior [27]. Similar behavior, termed critical behavior, has been observed in other systems, including cardiac pacemaker cells and GnRH secretion [28,29]. In the islet, critical behavior also leads to large decreases in electrical activity following small changes in the expression of ATP-insensitive mutant K_ATP_ channels. This occurs as a result of the suppressive effect subpopulations of inexcitable cells have in the islet [27]. Specifically a threshold number of inexcitable cells can be tolerated, beyond which electrical activity and insulin secretion is severely diminished. Importantly similar behavior occurs following uniform K_ATP_-channel activation with diazoxide, where some cells are less-excitable and some more excitable due to endogenous heterogeneity [30]. Such behavior was predicted by both a network model of islet cellular coupling [27,31], and a dynamic islet electrophysiological model [27,32], where in each case the overall islet electrical response is determined by the excitability of the constituent cellular population and the level of coupling. Consistent with predictions from these models, when Cx36 expression was reduced in the presence of ATP-insensitive mutant K_ATP_ expression, or diazoxide-mediated K_ATP_ activation, a recovery in islet [Ca^2+^] and insulin secretion was observed. This also prevented hyperglycemia and diabetes as a result of ATP-insensitive mutant K_ATP_ expression [33]. These findings imply that gap junction electrical coupling aids in the coordinated suppression of islet [Ca^2+^] and insulin release caused by mutations in *KCNJ11* and *ABCC8*. Thus a reduction of gap junction coupling in the islet may recover islet function and blunt diabetes caused by mutations in *KCNJ11* and *ABCC8*.

Another property of coupled multi-cellular systems is a lack of stochastic noise. At the single cell level stochastic behavior is observed in many cellular processes including gene transcription [34,35], and ion channel activity [36,37]. As discussed above, individual β-cells show heterogeneous function [30], with some cells showing reduced metabolic activity, excitability and insulin secretion compared to others [38–40]. However, isolated β-cells also show noisy, irregular fluctuations in membrane potential and [Ca^2+^] [41,42]. Ion channels show stochastic behavior where the channel switches rapidly between and open and closed states [36,43]. Previous work has demonstrated a strong effect of stochastic noise on [Ca^2+^] oscillations in uncoupled β-cells, but negligible effect in the presence of coupling [44]. In the absence of gap junction coupling stochastic behavior has been suggested to elevate [Ca^2+^] [33], and may therefore contribute to improved islet function in the presence of ATP-insensitive K_ATP_ channels.

In this study, we compared a computational model of islet electrical activity and insulin secretion against experimental measurements upon varied mutant K_ATP_ channel expression in the presence of different levels of gap junction coupling. Using this computational model, we then examined the role of gap junction coupling in exacerbating islet dysfunction in the presence of specific *KCNJ11* and *ABCC8* that cause NDM. We further examined the degree to which islet dysfunction can be overcome by reducing gap junction coupling, and the relative role of cellular heterogeneity and stochastic channel noise in mediating this.

## Results

### Progressive gap junction mediated recovery of [Ca^2+^] in model of NDM

**Fig 1:**
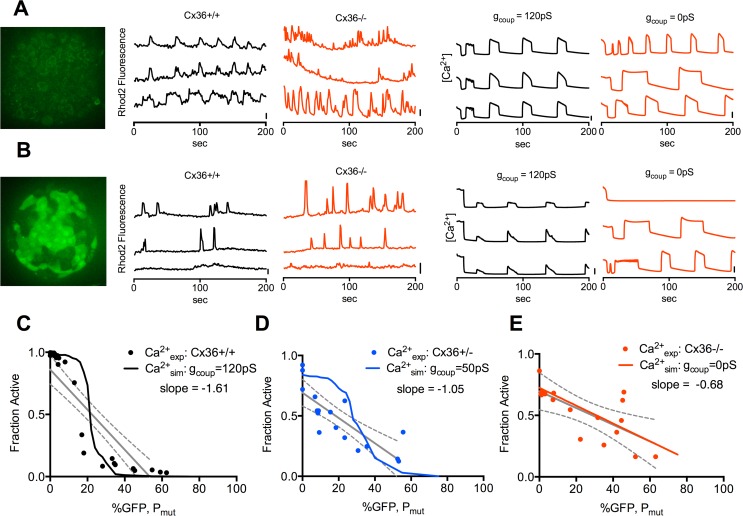
Calcium imaging and modeling in islets with varying Cx36 and Kir6.2^[ΔN30,K185Q]^ expression. **A).** Image of autofluorescence in GFP channel in islet lacking Kir6.2^[ΔN30,K185Q]^ expression (left); with representative time courses of Rhod2 fluorescence in islets isolated from Cx36^+/+^ and Cx36^-/-^ mice at 20mM glucose (middle); and time courses from simulations representing Cx36^+/+^ (*g*_*coup*_ = 120pS) and Cx36^-/-^ (*g*_*coup*_ = 0pS) islets at 20mM glucose (right). Scale bars represent 20% increase in Rhod2 fluorescence or 20% increase in simulated [Ca^2+^] respectively. **B).** As in A, for islets with ~50% Kir6.2^[ΔN30,K185Q]^ expression, as indicated by GFP fluorescence; or simulated to have ~50% Kir6.2^[ΔN30,K185Q]^ expression. **C)**. Quantification of [Ca^2+^], as expressed by the fraction of cells showing significant [Ca^2+^] elevations, in islets from Cx36^+/+^ mice with increasing Kir6.2^[ΔN30,K185Q]^ expression (pr with linear regression (solid grey line, dashed grey lines indicate 95% CI.); together with corresponding simulation results with *g*_*coup*_ = 120pS (solid black line). **D).** As in C, for islets from Cx36^+/-^ mice and simulation results with *g*_*coup*_ = 50pS. **E).** As in C, for islets from Cx36^-/-^ mice and simulation results with *g*_*coup*_ = 0pS. Each data point in C-E represents an average over n = 2–5 islets per mouse, with a total of N = 24 mice (Cx36^+/+^), 19 mice (Cx36^+/-^) or 22 mice (Cx36^-/-^).

β-cell specific expression of Kir6.2^[ΔN30,K185Q]^ under inducible Cre^ER^ control well-models human NDM [23,24,45,46]. To quantify how gap junction coupling exacerbates suppression of islet electrical activity following Kir6.2^[ΔN30,K185Q]^ expression we imaged [Ca^2+^] in islets of mice expressing variable levels of Kir6.2^[ΔN30,K185Q]^, as indicated by co-expression of GFP, for reduced levels of Cx36 (Fig 1A and 1B). Upon low levels of Kir6.2^[ΔN30,K185Q]^ expression, [Ca^2+^] elevation was observed across the islet, where the majority of β-cells showed frequent oscillations both in the presence and absence of Cx36 (Fig 1A). Upon high levels of Kir6.2^[ΔN30,K185Q]^ expression, few [Ca^2+^] transients were observed in the presence of Cx36, but these transients were more frequent, in more cells, in the absence of Cx36 (Fig 1B). Given heterogeneous cell responses, we quantified the proportion of cells showing significant elevations in [Ca^2+^] (which we now refer to as ‘[Ca^2+^]’). In the presence of normal gap junction coupling (Cx36^+/+^) a sharp transition was observed between high [Ca^2+^] (many cells showing elevations) and low [Ca^2+^] (few cells showing elevations) as Kir6.2^[ΔN30,K185Q]^ expression increased (Fig 1C) [27]. In the presence of ~45% coupling (Cx36^+/-^) the decline was less sharp, and right-shifted to higher Kir6.2^[ΔN30,K185Q]^ expression (Fig 1D). In the absence of coupling (Cx36^-/-^) the decline in [Ca^2+^] was gradual (Fig 1E). Regression analysis also showed the decline in [Ca^2+^] with increasing Kir6.2^[ΔN30,K185Q]^ become more gradual with reduced or absent gap junction coupling.

Previously, a computational model of islet electrical activity showed good agreement with experimental measurements of [Ca^2+^] upon increased Kir6.2^[ΔN30,K185Q]^ expression [27]. To further validate this model against our experimental measurements, we included the presence of variable Kir6.2^[ΔN30,K185Q]^-expressing cells and reduced electrical coupling (Fig 1A and 1B). Cx36^+/+^ (100% coupling) was represented by 120pS; Cx36^+/-^ (~45% coupling) was represented by 50pS; and Cx36^-/-^ (~0% coupling) was represented by 0pS [10,47]. In all cases there was good agreement between simulations and experimental measurements (Fig 1C–1E): Simulations representing Cx36^+/-^ showed a less rapid and right-shifted transition and simulations representing Cx36^-/-^ showed a linear decline, although the simulations generally showed a sharper transition than experimental measurements. Simulations also agreed with experimental data that above ~20% Kir6.2^[ΔN30,K185Q]^ expression near silent behavior was observed with normal levels of gap junction coupling, and a progressive increase in [Ca^2+^] occurred as gap junction coupling was reduced.

### Gap junction mediated recovery of insulin secretion and glucose homeostasis

**Fig 2:**
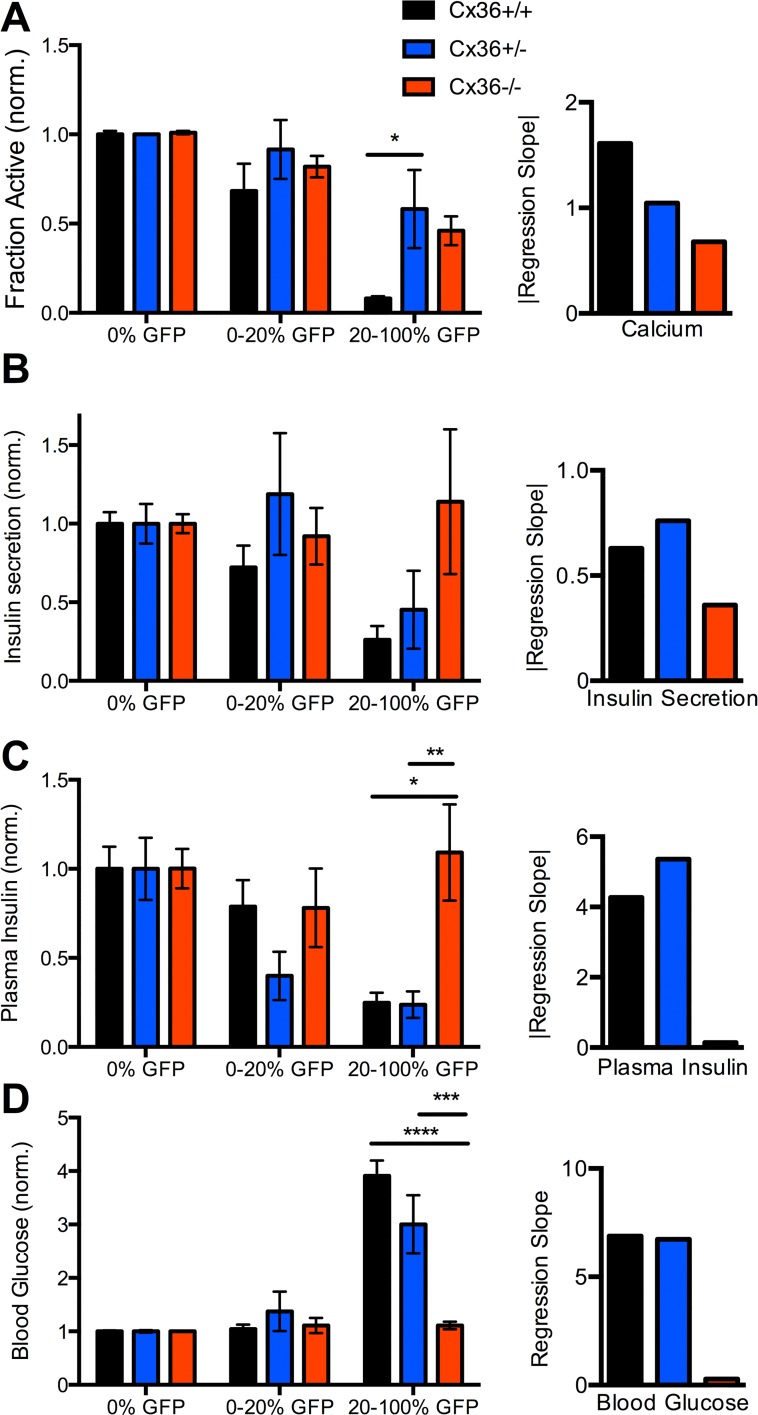
Physiological measurements with varying Cx36 and Kir6.2^[ΔN30,K185Q]^ expression. **A).** Left: [Ca^2+^] in isolated islets, as expressed by the fraction of cells showing significant [Ca^2+^] elevations; averaged over ranges of Kir6.2^[ΔN30,K185Q]^ expression, as defined by level of GFP expression, at 20mM glucose. Data is presented for the three levels of Cx36 expression (Cx36^+/+^—black; Cx36^+/-^—blue; Cx36^-/-^—red), normalized to the mean level in control islets with no Kir6.2^[ΔN30,K185Q]^ expression (0% GFP). Right: Linear regression slope for fraction of cells showing significant [Ca^2+^] elevation against %GFP, over the three levels of Cx36 expression. **B).** As in A for insulin secretion from isolated islets at 20mM glucose. **C).** As in A for plasma insulin levels under adlib feeding (day 29 post tamoxifen induction) in mice from which islets were isolated. **D).** As in A for blood glucose in mice under *ad lib* feeding from which islets were isolated. Data is presented as mean±s.e.m. for a total N = 24 mice (Cx36^+/+^), 19 mice (Cx36^+/-^) or 22 mice (Cx36^-/-^). *** (p < .0001), ***(p < .001), **(p < .01), *(p < .05) indicate significant differences between experimental groups as indicated. Comparison of multiple means was performed using ANOVA and Tukey’s HSD test.

We next examined whether the gap junction dependence of Kir6.2^[ΔN30,K185Q]^-induced suppression of [Ca^2+^] was linked to altered insulin secretion and glucose homoeostasis (S1 Fig). Measurements of [Ca^2+^], insulin secretion, plasma insulin and glucose were grouped according to the level of Kir6.2^[ΔN30,K185Q]^ expression. Upon low (<20%) Kir6.2^[ΔN30,K185Q]^ expression little decrease in [Ca^2+^] was observed for all Cx36 levels (Fig 2A), whereas upon high (>20%) Kir6.2^[ΔN30,K185Q]^ expression the reduction in [Ca^2+^] was significantly less for reduced Cx36 levels (Cx36^+/-^) or absent Cx36 (Cx36^-/-^) compared to normal Cx36 levels (Cx36^+/+^) (Fig 2A).

Insulin secretion and plasma insulin showed general agreement with [Ca^2+^] measurements (Fig 2B and 2C). Upon low (<20%) Kir6.2^[ΔN30,K185Q]^ expression little decrease was observed for all Cx36 levels, and upon high (>20%) Kir6.2^[ΔN30,K185Q]^ expression there was no reduction in insulin secretion and plasma insulin for Cx36^-/-^, compared to large reductions for Cx36^+/+^ (Fig 2B and 2C). However large reductions in plasma insulin and insulin secretion were also observed for Cx36^+/-^ (Fig 2B and 2C), although the variability was greater than Cx36^+/+^ (S1 Fig). Blood glucose showed good correspondence to plasma insulin and insulin secretion measurements (Fig 2D). Upon low (<20%) Kir6.2^[ΔN30,K185Q]^ expression, euglycemia was observed for all Cx36 levels. Upon high (>20%) Kir6.2^[ΔN30,K185Q]^ expression euglycemia was observed for Cx36^-/-^ but substantial hyperglycemia was observed for Cx36^+/+^ and Cx36^+/-^, albeit slightly less for Cx36^+/-^. Regression analysis showed similar strong variations in insulin secretion, plasma insulin levels and blood glucose with increasing Kir6.2^[ΔN30,K185Q]^ expression for Cx36^+/+^ and Cx36^+/-^, but less or absent variations for Cx36^-/-^ (Fig 2B–2D).

### Reduced ATP sensitivity triggers critical behavior in simulated islets

**Fig 3:**
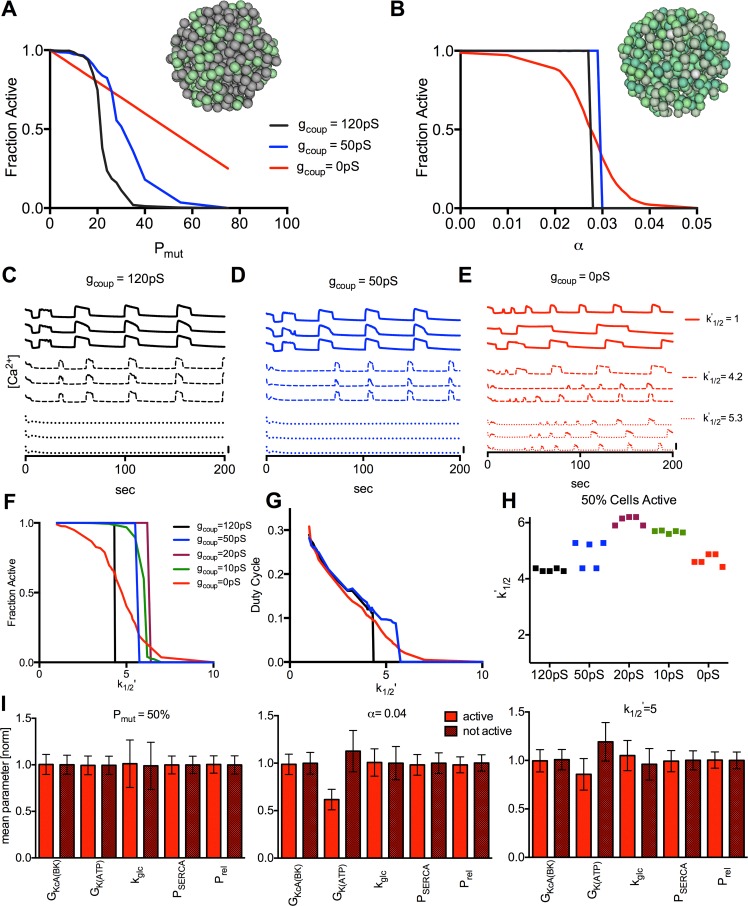
Simulated dependence of islet [Ca^2+^] on electrical coupling upon reduced K_ATP_ ATP sensitivity. **A).** Quantification of [Ca^2+^], as expressed by the fraction of cells showing significant [Ca^2+^] elevations, for simulations of Kir6.2^[ΔN30,K185Q]^ expression in a percentage of cells (*P*_*Mut*_), at 20mM glucose, with *g*_*coup*_ = 120pS (black), 50pS (blue), and 0pS (red). Inset represents islet where specific cells show increased K_ATP_ activity modelling mosaic Kir6.2^[ΔN30,K185Q]^ expression (green). **B).** Fraction of cells showing significant [Ca^2+^] elevations for simulations of diazoxide application uniformly in all cells (*α*) at 11mM glucose, *P*_*mu*t_ = 1, with *g*_*coup*_ as in A. Inset represents islet where cells show variable K_ATP_ activity modelling intrinsic heterogeneity (green). **C).** Representative time courses from simulations with increased *k*^*’*^_*1/2*_ values (reduced ATP-sensitivity) as indicated, far left for *g*_*coup*_ = 120pS. **D).** As in C for *g*_*coup*_ = 50pS. **E).** As in C for *g*_*coup*_ = 0pS. Scale bars represent 20% increase in simulated [Ca^2+^]. **F).** Fraction of cells showing significant [Ca^2+^] elevations for increased *k*^*’*^_*1/2*_ values uniformly in all cells at 11mM glucose, *P*_*mu*t_ = 1, for *g*_*coup*_ = 120pS (black), 50pS (blue), 20pS (purple), 10pS (green), and 0pS (red). **G).** Mean [Ca^2+^] duty cycle for increased *k*^*’*^_*1/2*_ values as in F. **H).** *k*^*’*^_*1/2*_ value at which 50% of cells show significant [Ca^2+^] elevations for values of *g*_*coup*_ over 5 simulated islets. **I).** Mean±SD cell parameters for active and inactive cells at *g*_*coup*_ = 0pS for simulations in A (*P*_*mut*_), B (*α*), F (*k*^*’*^_*1/2*_). All data is representative of n = 5 simulations with different random number seeds.

While Kir6.2^[ΔN30,K185Q]^ expression well-models NDM, mosaic expression of mutant K_ATP_ channels is unlikely in human disease. However a similar rapid onset of suppression also occurs upon increasing diazoxide activation of K_ATP_ channels across all β-cells, due to endogenous β-cell heterogeneity [27]. Similar behavior also occurred when simulating both a progressive increase in mosaic over-active K_ATP_ channels (P_mut,_ modelling Kir6.2^[ΔN30,K185Q]^) and uniform K_ATP_ activation (α, modelling diazoixde) (Fig 3A and 3B). A sharp transition occurred in each case in the presence of full electrical coupling, whereas with reduced electrical coupling a right-shifted transition occurred, which was less sharp for increasing P_mut_. In the absence of electrical coupling a further right-shifted and more gradual transition occurred in each case. Therefore similar behavior occurs for different ways in which reductions in excitability are introduced to the islet.

We predicted similar gap junction dependence would occur in the presence of over-active K_ATP_ channels resulting from mutations to *KCNJ11* and *ABCC8* that cause NDM. We simulated how altered ATP-inhibition kinetics (Eq 7) impact [Ca^2+^]. Several *KCNJ11* and *ABCC8* mutations have been reported to show a residual current at saturating ATP concentrations (*α*) [20,22] and all mutations are reported to show a shift in the half maximal ATP concentration for K_ATP_ inhibition (*k*^*’*^_*1/2*_) [14,16–21,48–56]. The decline in [Ca^2+^] upon increasing residual current (*α*) is equivalent to that in Fig 3B. Upon increasing the half maximal ATP concentration (*k*^*’*^_*1/2*_) there was also a decline in [Ca^2+^] (Fig 3C–3E). In the presence of full electrical coupling, all cells remained active until *k*^*’*^_*1/2*_ increased by ~4.3, at which point a sharp transition to full suppression occurred (Fig 3F). In the presence of progressively reduced electrical coupling, similar sharp transitions occurred but at greater *k*^*’*^_*1/2*_ values, which became less sharp at <15% electrical coupling (Fig 3F). In the absence of electrical coupling, a gradual transition was observed where many cells remained active for *k*^*’*^_*1/2*_ values up to ~10 (Fig 3F). The [Ca^2+^] duty-cycle showed similar behavior (Fig 3G), although no decrease occurred for absent electrical coupling at low *k*^*’*^_*1/2*_ values. Simulations at full electrical coupling were well-repeatable, but showed variable shifts in activity at reduced electrical coupling (Fig 3H). As such for <45% electrical coupling the transition became more gradual averaged over an ensemble of islets.

We then characterized the β-cells that showed elevated [Ca^2+^] in the absence of electrical coupling (Fig 3I). For increased P_mut_ (Fig 3A), cells with elevated [Ca^2+^] lacked Kir6.2^[ΔN30,K185Q]^ expression and were not distinguished by differences in other parameters. For increased α (Fig 3B), cells with elevated [Ca^2+^] had reduced *g*_*KATP*._ For increased *k’*_*1/2*_ (Fig 3F), cells with elevated [Ca^2+^] had reduced *g*_*KATP*_ and increased *k*_*glyc*_. Thus in the absence of coupling, cells that remained active had increased excitability, determined by K_ATP_ density or function, and metabolic activity.

**Fig 4:**
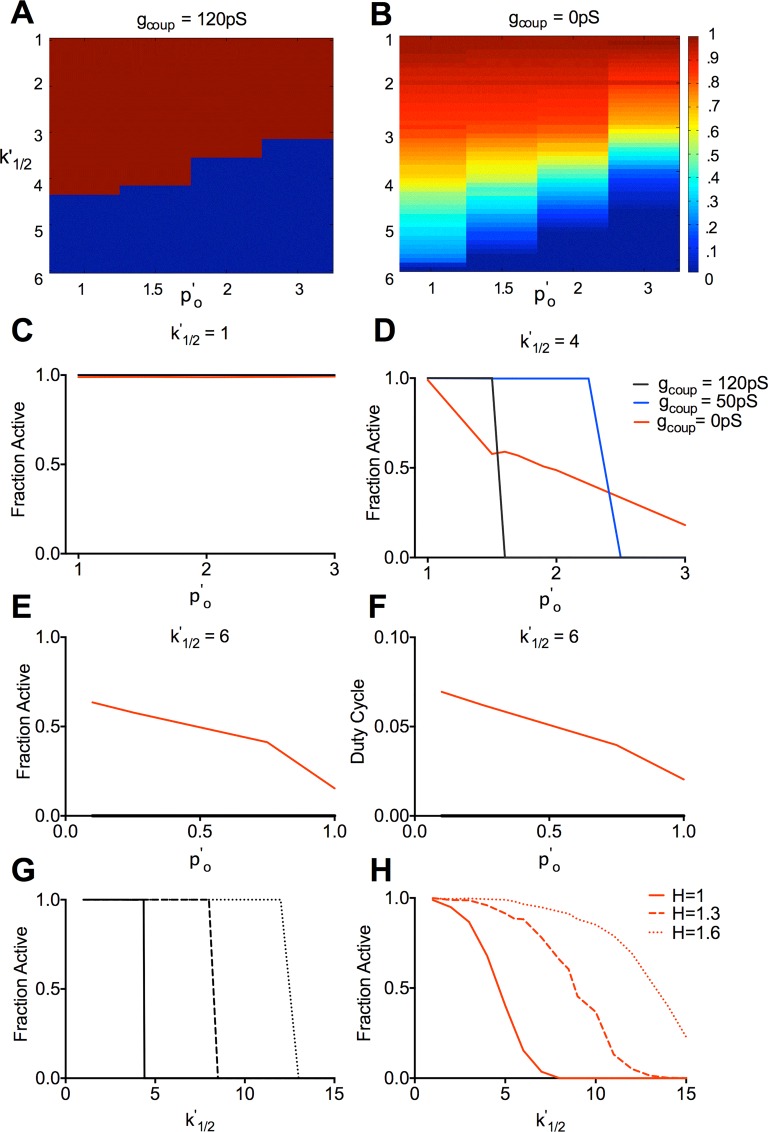
Simulated dependence of [Ca^2+^] on electrical coupling following modulation of other K_ATP_ parameters. **A).** False-color scale map displaying the fraction of cells showing significant [Ca^2+^] elevations for *g*_*coup*_ = 120pS upon variation in both *k*^*’*^_*1/2*_ (ATP-sensitivity) and *p*^*’*^_*o*_ (open channel conductance) values, as indicated. **B).** As in A, for *g*_*coup*_ = 0pS. **C).** Fraction of cells showing significant [Ca^2+^] elevations upon increased *p*^*’*^_*o*_ values at *k*^*’*^_*1/2*_ = 1, for *g*_*coup*_ = 120pS, 50pS, and 0pS. **D).** As in C for *k*^*’*^_*1/2*_ = 4. **E).** Fraction of cells showing significant [Ca^2+^] elevations upon decreased *p*^*’*^_*o*_ values at *k*^*’*^_*1/2*_ = 6 for *g*_*coup*_ = 120pS and 0pS. **F).** Mean [Ca^2+^] duty cycle upon decreased *p*^*’*^_*o*_ values at *k*^*’*^_*1/2*_ = 6, as in E. **G).** Fraction of cells showing significant [Ca^2+^] elevations for *g*_*coup*_ = 120pS upon increased *k*^*’*^_*1/2*_ values for different *H* values, as indicated. **H).** as in G for *g*_*coup*_ = 0pS. All simulations were run at 11mM glucose, *P*_*mu*t_ = 1. Data is representative of n = 5 simulations with different random number seeds.

Other parameters characterizing changes to K_ATP_ ATP-inhibition kinetics are also sometimes reported for *KCNJ11* and *ABCC8* mutations. The open channel probability in the absence of ATP (*p’*_*0*_) increases for a number of NDM mutations. We characterized how changes in both *p’*_*0*_ and *k*^*’*^_*1/2*_ act in combination, in the presence and absence of electrical coupling (Fig 4A and 4B). Increases in *p’*_*0*_ alone had little effect for any level of coupling (Fig 4C). Upon higher *k*^*’*^_*1/2*_ values, increases in *p’*_*0*_ led to a sharp transition to [Ca^2+^] suppression with normal electrical coupling, a shift in this transition with reduced electrical coupling, and a gradual decline in activity even at high *p’*_*0*_ values in the absence of coupling (Fig 4D). At high *k*^*’*^_*1/2*_ values where only a few cells are active in the absence of coupling, a decrease in *p’*_*0*_ elevated [Ca^2+^], but only in the absence of electrical coupling (Fig 4E and 4F). A change in the steepness of ATP inhibition (hill coefficient, *H*) is sometimes reported. Increasing *H* did not affect the dependence of the transition on electrical coupling (Fig 4G and 4H), although in each case the transition occurred at higher *k*^*’*^_*1/2*_ values. Increasing *H* lowers *po*_*K*(*ATP*)_ within the range of cellular ATP levels, which are substantially higher than the half maximal ATP concentration for K_ATP_ inhibition.

These results demonstrate that similar behavior occurs irrespective of the way in which K_ATP_ over-activity is increased in the islet (S2 Fig): a sharp decline in activity in the presence of electrical coupling; but a shifted and/or more gradual decline in the absence of electrical coupling.

### Simulated Kir6.2, SUR1 mutations show gap junction mediated recovery of [Ca^2+^]

We next simulated specific *KCNJ11* and *ABCC8* mutations that cause NDM, and correlated the suppression of [Ca^2+^] with the clinical severity of the mutation (type2 diabetes; transient or permanent NDM; DEND characteristics) that indicates the level of islet dysfunction associated with each mutation. In each mutation we further tested whether a recovery in [Ca^2+^] could be achieved by eliminating electrical coupling. All mutations in which ATP-sensitivity is characterized (Eq 7) report shifts in *k*^*’*^_*1/2*_. *p’*_*0*_ when reported is also elevated, and occasionally *α* is characterized and reported as non-zero (S1 Table). However *p’*_*0*_ and *α* are often not reported, or are assumed. We first examined a set of *KCNJ11* and *ABCC8* mutations, as characterized by reported *k*^*’*^_*1/2*_ and *α* values for channels composed of mixed mutant and wild-type Kir6.2 or SUR1 subunits. A review of NDM mutations has reported K_ATP_ currents at elevated, but not saturating ATP levels [57]. Given reported *k*^*’*^_*1/2*_ values, we then estimated a residual open probability (*α*_*est*_, S1 Table). Therefore we also examined a set of *KCNJ11* and *ABCC8* mutations as characterized by reported *k*^*’*^_*1/2*_ and estimated *α*_*est*_ values.

**Fig 5:**
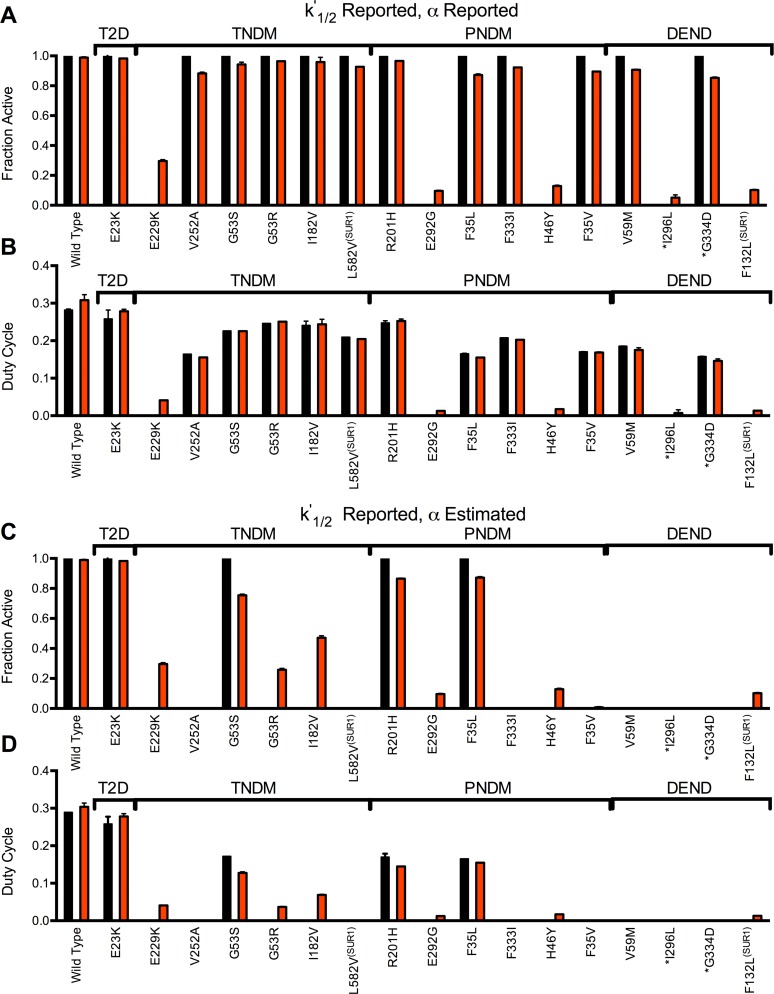
Simulated decline in [Ca^2+^] due to Kir6.2 and SUR1 mutations, and recovery following decreased electrical coupling. **A).** Fraction of cells showing significant [Ca^2+^] elevations for simulations that include mutant K_ATP_ channel activity, for *g*_*coup*_ = 120pS (black) and *g*_*coup*_ = 0pS (red). Simulations include the characterized mutations indicated where reported *k*^*’*^_*1/2*_ changes and reported *α* (if any) are accounted for. **B).** Mean [Ca^2+^] duty cycle for simulations that include mutant K_ATP_ channel activity as in A. **C)**. Fraction of cells showing significant [Ca^2+^] elevations for simulations that include mutant K_ATP_ channel activity, for *g*_*coup*_ = 120pS (black) and *g*_*coup*_ = 0pS (red). Simulations include the characterized mutations indicated where reported *k*^*’*^_*1/2*_ changes and estimated *α* (see S2 Table) are accounted for. **D).** Mean [Ca^2+^] duty cycle for simulations that include mutant K_ATP_ channel activity as in C. All simulations were run at 11mM glucose, *P*_*mu*t_ = 1. Data is presented as mean±SD for n = 3–5 simulations with different random number seeds. Results are arranged in order of clinical severity, with the clinical classification indicated: T2D- (Type2 Diabetes; TNDM- Transient Neonatal Diabetes Mellitus; PNDM- Permanent Neonatal Diabetes Mellitus; DEND- PNDM with Developmental Delay and Neurological features, including iDEND. * indicates mutations where sulfonylurea therapy is reported to be ineffective.

When including reported *k*^*’*^_*1/2*_ and reported *α* values, simulated islets showed reduced [Ca^2+^] in the presence of electrical coupling (Fig 5A and 5B, S3 Fig). The majority of mutations showed all cells with [Ca^2+^] elevations (Fig 5A and S3 Fig), but reduced [Ca^2+^] duty cycle (Fig 5B and S3 Fig). The [Ca^2+^] duty cycle was reduced to a greater level in all NDM mutations compared to the type2 diabetes mutation, owing to the greater *k*^*’*^_*1/2*_. However, in the majority of mutations the [Ca^2+^] duty cycle was still >50% of that in control islets. Complete suppression of [Ca^2+^] was observed for only a subset of mutations across all clinical characterizations of NDM. In the absence of electrical coupling, recovery of [Ca^2+^] was observed in the majority of these cases. This included sulfonylurea-insensitive mutations, such as I296L that had no reported residual current (*α*).

When *α*_*est*_ values were included, most NDM mutations showed complete suppression of [Ca^2+^] (Fig 5C and 5D, S4 Fig), albeit with some exceptions (e.g. DEND mutation V64L). In the absence of coupling, many mutations showed a partial recovery in [Ca^2+^]. This included the majority of transient NDM mutations, but also several permanent NDM mutations (E229G, H46Y, F35V) and those with DEND features (F132L) (Fig 5C and S4 Fig). Thus accounting for a residual open probability (*α*) is important to describe the action of *KCNJ11* and *ABCC8* mutations; and in many cases a recovery in islet function is predicted following a modulation of gap junction electrical coupling.

### Stochastic channel noise increases [Ca^2+^] in the absence of gap junction coupling

**Fig 6:**
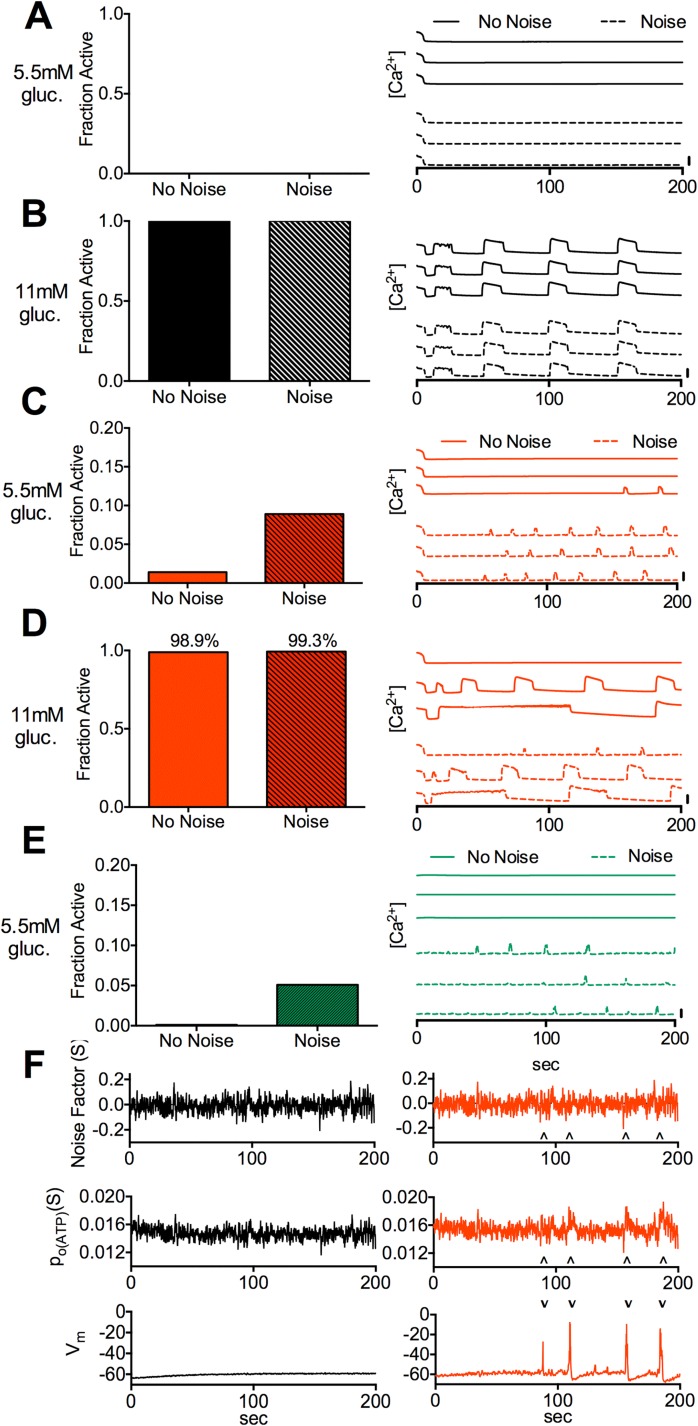
Simulated effect of stochastic channel noise at basal and stimulatory glucose conditions. **A).** Fraction of cells showing significant [Ca^2+^] elevations (left) and representative time-courses of [Ca^2+^] (right) for simulations at 5.5mM glucose with g_coup_ = 120pS, with and without stochastic channel noise. **B).** As in A for simulations at 11mM glucose and g_coup_ = 120pS. **C).** As in A for simulations at 5.5mM glucose and *g*_*coup*_ = 0pS. **D).** As in A for simulations at 11mM glucose and *g*_*coup*_ = 0pS. **E).** As in A for simulations at 5.5mM glucose and *g*_*coup*_ = 10pS. Vertical scale bars represent 20% increase in simulated [Ca^2+^]. **F).** Time courses of K_ATP_ noise factor (*S*), K_ATP_ open probability (*p*_*0(KATP)*_) and membrane potential (*V*_*m*_) in a representative cell for *g*_*coup*_ = 120pS (left) and *g*_*coup*_ = 0pS (right). Arrows indicate excursions in *S* and *p*_*0(KATP)*_ that correspond to substantial membrane depolarization. All simulations were run with *P*_*mu*t_ = 1. Data in A,C,E is presented as mean for n = 10 simulations with different random number seeds.

We next accounted for the stochastic nature of ion channels by first simulating islets with normal K_ATP_ function and testing the effects of stochastic channel noise at basal and stimulatory glucose. In the presence of electrical coupling, including noise resulted in no change in [Ca^2+^] at basal and elevated glucose (Fig 6A and 6B). In the absence of electrical coupling, including noise resulted in more cells showing [Ca^2+^] elevations at basal glucose (Fig 6C). Minor increases in cells showing [Ca^2+^] elevations were also observed at elevated glucose (Fig 6D), with small changes in [Ca^2+^] oscillation shape and duty cycle. At ~8% electrical coupling including noise also resulted in more cells showing [Ca^2+^] elevations at basal glucose (Fig 6E). [Ca^2+^] elevations at basal glucose were characterized by short transient peaks (Fig 6C), similar to those experimentally observed in Kir6.2^[ΔN30,K185Q]^ positive cells lacking gap junction coupling [33]. Transient K_ATP_ closures were observed in the presence and absence of coupling, but only in the absence of electrical coupling did these coincide with significant depolarization and [Ca^2+^] elevations (Fig 6F). Thus stochastic noise leads to increases in [Ca^2+^] under basal glucose, upon reduced or absent electrical coupling.

**Fig 7:**
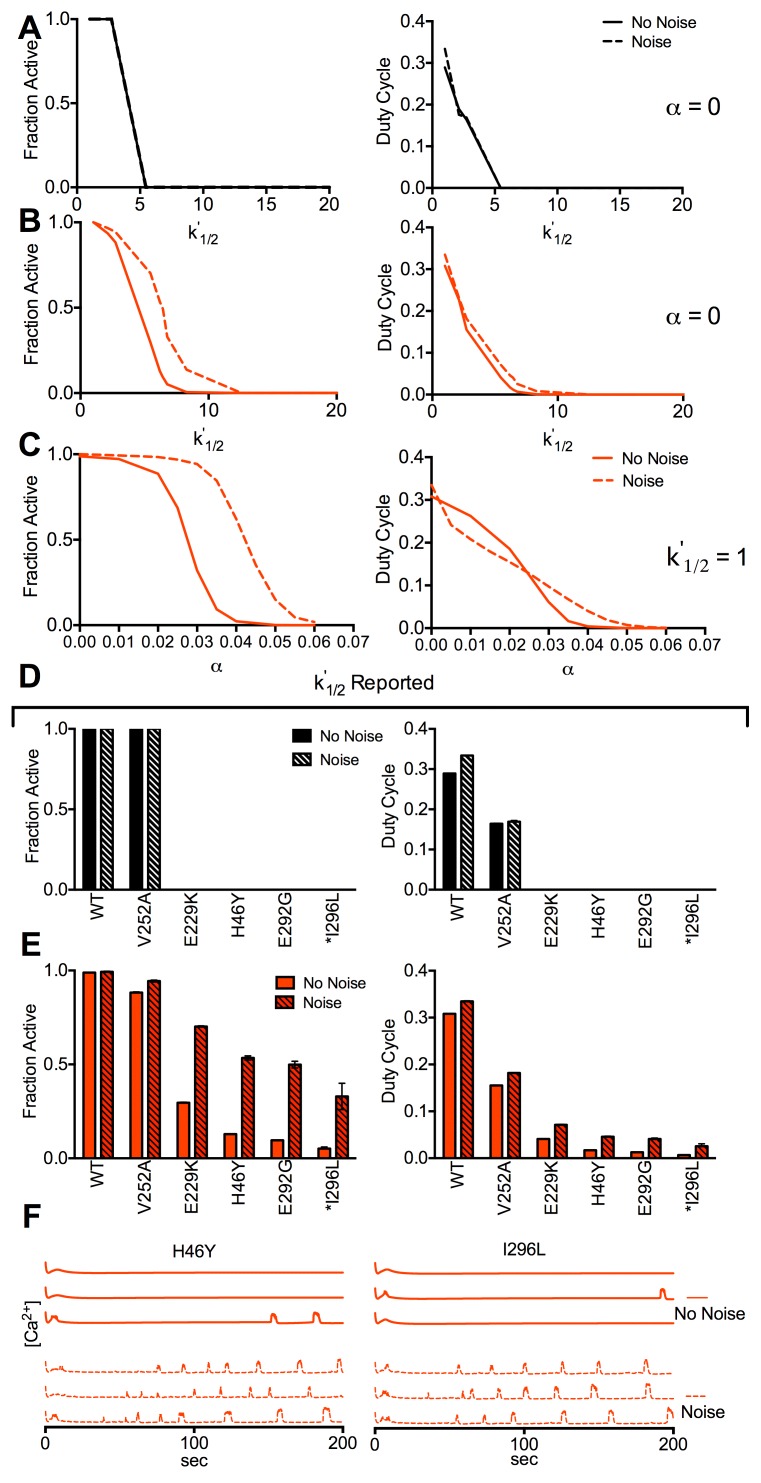
Simulated effect of stochastic channel noise upon Kir6.2 and SUR1 mutations. **A).** Fraction of cells showing significant [Ca^2+^] elevations (left) and mean [Ca^2+^] duty cycle (right), for simulations with and without stochastic channel noise, for increasing *k*^*’*^_*1/2*_ with *g*_*coup*_ = 120pS (*α* = 0). **B).** As in A for increasing *k*^*’*^_*1/2*_ with *g*_*coup*_ = 0pS (*α* = 0). **C).** As in A for increasing *α* with *g*_*coup*_ = 0pS (*k*^*’*^_*1/2*_ = 1). **D).** Fraction of cells showing significant [Ca^2+^] elevations (left) and mean [Ca^2+^] duty cycle (right), for simulations with and without stochastic channel noise, that include mutant K_ATP_ channel activity for *g*_*coup*_ = 120pS. Simulations include the characterized mutations indicated where reported *k*^*’*^_*1/2*_ changes and reported *α* (if any) are accounted for. **E).** As in D for simulations with and without stochastic channel noise, that include mutant K_ATP_ channel activity for *g*_*coup*_ = 0pS. **F).** Representative time courses for simulations as in E, with and without stochastic channel noise for *g*_*coup*_ = 0pS. Vertical scale bars represent 20% increase in simulated [Ca^2+^]. All simulations were run at 11mM glucose, *P*_*mu*t_ = 1. Data in D,E is presented as mean±SD for n = 3–5 simulations with different random number seeds.

We next examined the effect of stochastic noise in islets with altered K_ATP_ ATP-inhibition kinetics, focusing on increased *k*^*’*^_*1/2*_ and *α* values. In the presence of electrical coupling noise did not change how [Ca^2+^] depended on increasing *k*^*’*^_*1/2*_ (Fig 7A). However in the absence of electrical coupling noise enhanced [Ca^2+^] elevations at increasing *k*^*’*^_*1/2*_ (Fig 7B) and increasing *α* (Fig 7C), and elevated [Ca^2+^] up to higher *k*^*’*^_*1/2*_ or *α* values. We then asked if recoveries in [Ca^2+^] would be enhanced for the *KCNJ11* and *ABCC8* mutations we previously examined. For mutations described by reported *k*^*’*^_*1/2*_ and reported *α* where a recovery was predicted, in the presence of electrical coupling [Ca^2+^] remained fully suppressed when noise was included (Fig 7D). In the absence of electrical coupling [Ca^2+^] elevations were much greater when noise was included (Fig 7E). These elevations were again characterized by frequent, low duty cycle [Ca^2+^] transients (Fig 7F). Similarly, for those mutations described by reported *k*^*’*^_*1/2*_ and estimated *α* where a recovery was predicted, in the absence of electrical coupling [Ca^2+^] elevations were much greater when noise was included (S5 Fig). Thus after accounting for stochastic noise a much greater recovery in islet function is predicted following a reduction in gap junction coupling.

### Suppression of insulin secretion and gap junction mediated recovery

**Fig 8:**
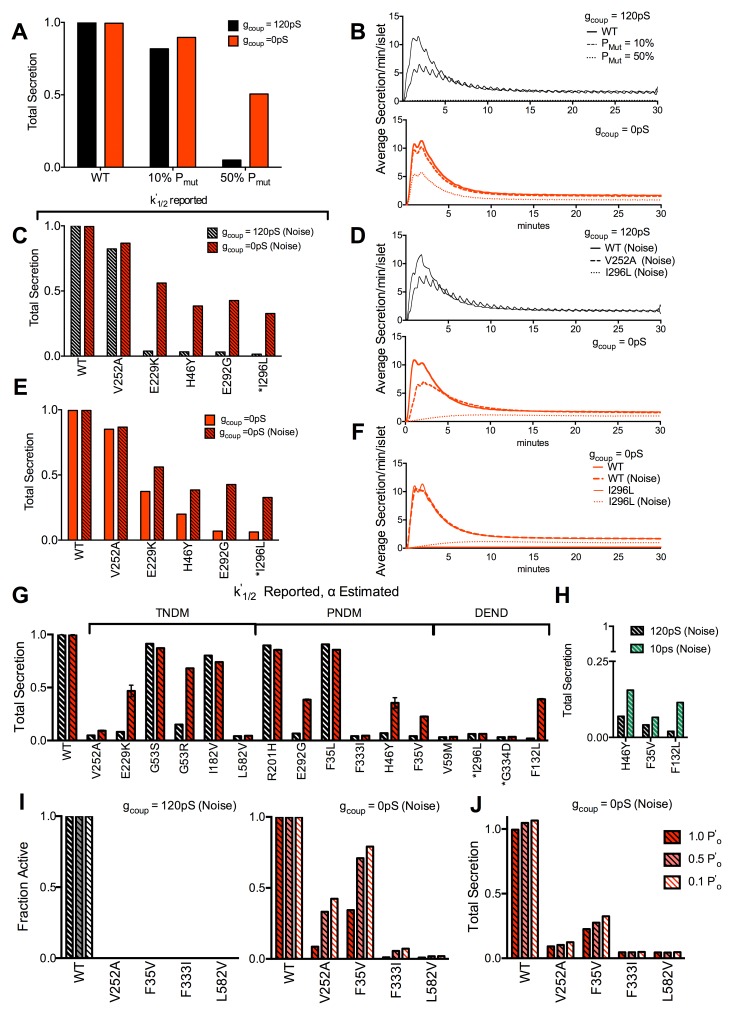
Simulated decline in insulin secretion due to Kir6.2 and SUR1 mutations. **A).** Total time-averaged insulin secretion for simulations of Kir6.2^[ΔN30,K185Q]^ expression in a fraction of cells indicated (*P*_*Mut*_), with *g*_*coup*_ = 120pS (black), and 0pS (red). **B).** Representative time-courses of insulin secretion averaged across the simulated islet for each *P*_*Mut*_ and *g*_*coup*_ in A. **C).** Total time-averaged insulin secretion for simulations that include mutant K_ATP_ channel activity for *g*_*coup*_ = 120pS and *g*_*coup*_ = 0pS, with stochastic channel noise. Simulations include the characterized mutations indicated where reported *k*^*’*^_*1/2*_ changes and reported *α* (if any) are accounted for. **D).** Representative time-courses of insulin secretion averaged across the simulated islet for indicated mutations and *g*_*coup*_ in C. **E).** Total time-averaged insulin secretion for simulations that include mutant K_ATP_ channel activity, as in C, with and without stochastic channel noise for *g*_*coup*_ = 0pS. **F).** Representative time-courses of insulin secretion averaged across the simulated islet for indicated mutations and noise in E. **G).** Total time-averaged insulin secretion for simulations that include mutant K_ATP_ channel activity for *g*_*coup*_ = 120pS and *g*_*coup*_ = 0pS, with stochastic channel noise. Simulations include the characterized mutations indicated where reported *k*^*’*^_*1/2*_ changes and estimated *α* are accounted for. **H).** As in G for *g*_*coup*_ = 120pS and *g*_*coup*_ = 10pS. **I).** Fraction of cells showing significant [Ca^2+^] elevations for simulations that include selected mutant K_ATP_ channel activity, as in G, for *g*_*coup*_ = 120pS (left) and *g*_*coup*_ = 0pS (right) and with stochastic channel noise, as *p’*_*0*_ is reduced to model sulfonylurea action. **J).** Total time-averaged insulin secretion for simulations as in H, that include selected mutant K_ATP_ channel activity, as in G, for *g*_*coup*_ = 0pS with stochastic channel noise, as *p’*_*0*_ is reduced to model sulfonylurea action. Results are arranged in order of clinical severity, with the clinical classification indicated in G. * indicates mutations where sulfonylurea therapy is reported to be ineffective. Simulations in A,B were run at 20mM glucose, where all others were run at 11mM glucose, *P*_*mu*t_ = 1. Data in A,C,E,G,H,I is presented as mean±SD for n = 3 simulations with different random number seeds.

To predict how a reduction in gap junction coupling may recover insulin secretion in the presence of *KCNJ11* or *ABCC8* mutations, we included an insulin-release component to the islet model [58]. In control islets a step elevation of glucose or [Ca^2+^] generated biphasic secretion dynamics (S6 Fig) consistent with experimental observations [59–61]. To verify the islet model can accurately predict gap junction recovery of insulin release (Fig 2B) we first simulated islets with increasing Kir6.2^[ΔN30,K185Q]^ expression. Time-averaged insulin secretion was similar for control islets in the presence and absence of electrical coupling (Fig 8A). Upon 10% over-active K_ATP_ channels there was a small decline in insulin release in each case, restricted to the first phase, t = 0-5min (Fig 8B). Upon 50% over-active K_ATP_ channels there was a substantial decline in insulin release in the presence of electrical coupling, close to residual levels. However, in the absence of electrical coupling the decline was less, consistent with observations here (Fig 2B and S1 Fig) and published elsewhere [33].

We next examined if a reduction in electrical coupling recovered insulin secretion in the presence of the *KCNJ11* and *ABCC8* mutations that we predicted would show [Ca^2+^] recovery (Figs 5 and 7, S5 Fig). When considering mutations described by reported *k*^*’*^_*1/2*_ and reported *α* (if any), those mutations that showed recoveries in [Ca^2+^] (Figs 5A, 7B, 7D and 7E) also showed recoveries in insulin secretion (Fig 8C). In the presence of electrical coupling the suppression in insulin secretion was much greater in the first phase (t = 0-5min) than the second phase (t>5min) (S7 Fig). The recovery of insulin secretion in the absence of coupling was also greater in the second phase (Fig 8D and S7 Fig), primarily due to the slow [Ca^2+^] elevation limiting significant [Ca^2+^] and insulin secretion in the first phase (S7 Fig). Stochastic noise had a greater effect in promoting recovery for more severe mutations, e.g. I296L (Fig 8E and 8F). When considering mutations described by both reported *k*^*’*^_*1/2*_ and estimated *α*, those mutations that showed recoveries in [Ca^2+^] (Fig 5C and 5D, S5 Fig) also showed recoveries in insulin secretion (Fig 8G). A sub-set of these mutations also showed recoveries in [Ca^2+^] and insulin secretion at ~8% electrical coupling (Fig 8H and S5 Fig).

Sulfonylureas can achieve near-full inhibition of most mutant K_ATP_ channels, albeit with complex inhibition kinetics. By reducing *p’*_*0*_ we modeled the effect of sulfonylurea action and applied this to *KCNJ11* and *ABCC8* mutations described by reported *k*^*’*^_*1/2*_ and estimated *α* that showed little or no [Ca^2+^] recovery (S8 Fig). In the presence of electrical coupling no increase in [Ca^2+^] occurred upon *p’*_*0*_ reduction for all mutations examined (Fig 8I). In the absence of electrical coupling a significant recovery in [Ca^2+^] (Fig 8I) and insulin secretion (Fig 8J) occurred upon *p’*_*0*_ reduction for several of the mutations examined. Thus further recoveries are predicted for severe NDM mutations, with decreased or absent gap junction coupling upon sulfonylurea therapy.

## Discussion

The islet of Langerhans shows global coordinated behavior as a result of electrical coupling between β-cells [9,10]. β-cells are intrinsically heterogeneous in their function and previous work demonstrated how a threshold number of inactive cells can suppress the activity in cells that otherwise would be active, mediated by electrical coupling [27]. Factors that reduce β-cell excitability through increasing K_ATP_ channel activity will lead to there being a greater number of inactive β-cells in the islet and thus a greater likelihood of overall islet suppression. Thus reducing electrical coupling may prevent this suppression by limiting it to just those inactive cells. In this study we used experimental and computational methods to examine how electrical coupling between β-cells can exacerbate islet dysfunction following changes to K_ATP_ channel activity due to *KCNJ11* and *ABCC8* mutations that cause NDM; and that modulating electrical coupling may recover function.

### Electrical coupling between β-cells mediates phase transition in global islet function

We previously predicted with a mathematical model how the sharp transition to global suppression that occurs upon small increases in the number of inexcitable cells would become more gradual upon reduced electrical coupling [27]. Here we experimentally demonstrate this with good quantitative agreement (Fig 1): With ~45% gap junction coupling a less sharp and right-shifted transition is observed, and in the absence of gap junction coupling there is a near-linear relationship where only excitable cells are active. With high proportions of inexcitable cells there is a progressive increase in electrical activity with reduced coupling. This increase arises from the remaining excitable cells being no longer suppressed via gap junction coupling. However, as we discuss below stochastic noise also plays a role in the absence of electrical coupling. We also show that these model predictions and experimental results extend to insulin secretion, which demonstrates how this behavior is physiologically important.

Through computational modeling we find similar sharp transitions to global suppression when parameters that describe the kinetics of K_ATP_ channel inhibition are uniformly changed across the islet (Figs 3 and 4). Previously similar behavior was shown computationally and experimentally upon uniform K_ATP_ activation with diazoxide [27]. These results support that irrespective of how inexcitability arises, electrical coupling will mediate the suppression of global activity; and that gradual reductions in gap junction coupling will increase activity. Furthermore the point at which the rapid transition to suppression occurs is consistent: For *P*_*mut*_ (% cells inactive), *α* (residual current), *k’*_*1/2*_ (ATP concentration at 50% K_ATP_ inhibition), *p’*_*0*_ (open channel probability), the transition in the presence of coupling occurs where 20–40% of cells are inactive in the absence of coupling.

We do find a greater variability in the transition position when simulating intermediate levels of electrical coupling compared to normal levels of coupling (Fig 3H). Further, in experimental measurements upon higher levels of Kir6.2^[ΔN30,K185Q]^ expression there was more variability between Cx36^+/-^ animals compared to Cx36^+/+^ animals (Fig 1D). This variability between islets may explain the less sharp transition we observed experimentally compared to simulations (Fig 1C–1E). This variability in the transition is also similar to the substantial variability in oscillation synchronization at stimulatory glucose previously observed experimentally in islets with ~50% gap junction coupling [8]. We speculate this variability in the transition depends on the link between heterogeneity in electrical coupling and cellular excitability, and whether the two correlate or not. Interestingly we observed further shifts in the transition at lower (<25%) levels of electrical coupling (Fig 3F), suggesting that a few remaining excitable cells can still ‘recruit’ other cells to become active at these low levels of coupling. Examining the role of cellular heterogeneity and understanding the divergence in islet function at lower levels of electrical coupling will be a goal for future work.

Studies here have only considered mouse islets. However there are some differences between mouse and human islets that need considering to fully translate these findings to human islet function. The architecture of human islets is different from that of mouse islets [62–64], and the cellular regulation of excitability has some differences [65]. Despite these differences the level of electrical coupling between β-cells is similar to that in mouse islets [66,67], and mediated by the same gap junction connexins [7]. In 3D β-cell structures there is also little size-dependence to suppression [31]. Human islets have been suggested to be made up of folded ‘sheets’ of β-cells [64], but large 2D structures show similar suppression to 3D structures [31]. Thus we predict similar behavior would be observed in human islets. This prediction is supported by consistency between simulations of *KCNJ11* and *ABCC8* mutations (with α_est_) and clinical diabetes severity (discussed below). However, determining whether phase transitions occur upon increasing K_ATP_ over-activity in human islets and their dependence on gap junction coupling, as well as testing whether a human β-cell based islet model can recapitulate these findings is needed.

Connectivity is crucial for dictating the overall dynamics of the islet and we predict that the concepts found in the islet can be applied to other systems relying on signaling by means of gap junction channels. For example, in brain injuries, gap junction coupling can disrupt [Ca^2+^] dynamics and increase cell death in interconnected cells [68]. The absence of gap junctions in these situations does not lead to [Ca^2+^] dysregulation and reduces cell death [68]. This is similar to results presented here, where gap junction removal specifically alleviates the consequences of diabetes causing mutations. In another example, pulsatile release of GnRH from remodeled GnRH neurons initiates puberty [69,70]. GnRH neurons depolarize in response to GABA before maturation, but switch during maturation to a hyperpolarizing response. This switch depends on the opening of the GABA channel [28] and is analogous to K_ATP_ channel closure observed in the β cell. We therefore speculate electrical coupling is important for this sharp transition, and therefore subtle changes in channel kinetics induces critical-like behavior in coupled systems.

### Gap junction mediated recovery of function is predicted in NDM

A major goal of this study was to predict the role gap junction coupling plays in the presence of *KCNJ11* and *ABCC8* mutations that cause NDM. Our initial computational model results show good agreement with experimental results presented here and published elsewhere regarding the suppression of both [Ca^2+^] and insulin upon elevated Kir6.2^[ΔN30,K185Q]^ expression, and its recovery upon reduced gap junction coupling (Figs 1C–1E, 2A–2C, and 8A)[27,33]. There is also good agreement between computational model results and published experimental measurements of diazoxide-induced suppression upon reduced gap junction coupling (Fig 3B) [27]. This validation of the model gives us confidence that robust predictions can be made for the role of gap junction coupling upon altered K_ATP_ activity caused by *KCNJ11* and *ABCC8* mutations. A summary of predictions over all conditions tested is shown in S2 Table.

Including published kinetics of ATP-inhibition for *KCNJ11* and *ABCC8* mutations in the model showed disruptions to [Ca^2+^] and insulin secretion (Figs 5 and 8). *k'*_*1/2*_ was important to describe this disruption and is widely reported. However few studies reported the residual current *α*, and [Ca^2+^] was highly dependent on this parameter (Fig 3F). Indeed without inclusion of *α*_est_ the decrease in [Ca^2+^] was minor and similar across all clinical phenotypes (Fig 5A and 5B), therefore is unlikely to be a full description of islet dysfunction in NDM. With estimates for *α* included [Ca^2+^] was generally suppressed across NDM mutations irrespective of clinical characteristics, but not for type2 diabetes mutations (Fig 5C and 5D). This also indicates the importance for characterizing and reporting the residual ATP-independent current when interpreting channel mutation characteristics. While the hill factor was also important for [Ca^2+^] suppression, it did not correlate with NDM and is not often reported. Despite the good agreement with estimates for *α* included, there were some NDM exceptions where altered ATP inhibition was insufficient to generate a full suppression of [Ca^2+^] and insulin that would cause diabetes. *KCNJ11* mutations that alter channel trafficking can cause hyperinsulinism [71], and trafficking alterations may be involved in NDM mutations. Altered expression of Kir6.2 and SUR1 subunits has also been observed for some NDM mutations, and may also contribute to altered islet function [72]. In recombinant systems where mutant channels are characterized, expression and trafficking can be variable [73]. Therefore expression of mixed mutant and WT channel subunits may not fully replicate in-vivo conditions, where increased expression of the mutant subunit may lead to increased K_ATP_ over-activity than in recombinant systems.

For several *KCNJ11* and *ABCC8* mutations we predicted significant recovery in [Ca^2+^] and insulin secretion with an elimination of electrical coupling (Fig 5). Given the agreement between predicted [Ca^2+^], insulin and clinical phenotype (Figs 5C, 5D and 8G), together with the model validation (Figs 1, 2 and 8A), we can have confidence that a reduction in gap junction coupling would be effective in recovering islet function for these *KCNJ11* and *ABCC8* mutations. The agreement between simulations of Kir6.2^[ΔN30,K185Q]^ expression, and *α* or *k’*_*1/2*_ increases (Fig 3); and experimentally measured recovery in insulin secretion and glucose homeostasis upon Kir6.2^[ΔN30,K185Q]^ expression with reduced gap junction coupling (Fig 2C and 2D), further supports these predictions. Recoveries were observed in most transient-NDM mutations, several permanent-NDM mutations, and few DEND mutations; although the latter heavily depends on whether *α*_*est*_ is included. For instance the sulfonylurea-insensitive I296L mutation showed no residual current in its initial characterization [21], yet based on other studies we estimate a significant residual current. The recovery in this and other DEND cases may be under- or over-estimated without determining whether there is a significant residual current. Nevertheless, these results indicate the importance of gap junction coupling in mediating the suppression of [Ca^2+^] and insulin secretion in NDM, and suggest reducing gap junction coupling may partially recover islet function and blunt NDM.

Most mutations required a complete reduction in electrical coupling to show recoveries in [Ca^2+^] and insulin secretion, although some mutations showed recovery with ~90% reduction. Achieving such large reductions in islet gap junction coupling has not been demonstrated using specific inhibitors, although strategies exist for robustly inhibiting gap junctions formed from other connexins [74]. Furthermore, reducing gap junction coupling will abolish coordinated dynamics of insulin release [60]. Despite increased [Ca^2+^] and insulin secretion, lack of pulsatility will likely reduce insulin action [75]. The absence of substantial first phase secretion recovery (S7 Fig) may have a similar impact. However chronic sulfonylurea delivery causes glucose intolerance [76], therefore any defect resulting from reduced gap junction coupling may not be more disruptive than long term sulfonylurea therapy.

We also did not include ‘amplifying’ mechanisms of insulin secretion or incretin (GLP1, GIP) hormone action, which would elevate Ca^2+^-triggered second-phase insulin secretion [77]. Incretin regulation of electrical activity and [Ca^2+^] has been simulated [78]; but amplifying mechanisms are poorly understood [61,79]. We predict for mutations where a minor second phase recovery occurs that a greater recovery will occur if we consider these mechanisms.

We predicted further recovery would occur in some permanent-NDM and DEND mutations with a combination of gap junction reduction and sulfonylurea treatment. However, for some mutations where no recovery was predicted, effective sulfonylurea therapy does occur in patients. We did not model sulfonylurea inhibition of the residual current *α*, which can explain this discrepancy. The kinetics of sulfonylurea inhibition of ATP-insensitive K_ATP_ channels is complex and not fully understood [80,81]. For example in-vitro studies have shown that the ‘sulfonylurea-insensitive’ I296L mutation is inhibited >50% under conditions of ~95% WT K_ATP_ inhibition. Whether the remaining current is ATP sensitive is unknown and will likely determine whether gap junction mediated recovery is possible: here we model it to be ATP-insensitive and thus present the minimal effectiveness expected. Therefore, while applicable to a rare patient population we predict that gap junction reduction may assist in sulfonylurea therapy for some insensitive or partially sensitive NDM mutations.

### Importance of stochastic noise to electrically isolated cell function

Another key finding is the role of stochastic noise to elevate electrical activity, [Ca^2+^] and insulin secretion in electrically isolated cells. While stochastic noise affects β-cell [Ca^2+^] oscillations [43,44], its role in basal [Ca^2+^] has not been examined significantly. We demonstrated noise can affect [Ca^2+^] levels (Fig 6). Including stochastic noise increased [Ca^2+^] only in the absence of electrical coupling at basal glucose, and upon increased K_ATP_ activity due to increased *α* and *k’*_*1/2*_. This suggests its broad importance in increasing activity in cells that lack electrical coupling. While we predict that stochastic noise will improve the recovery for several mutations, we note that for normal islets noise will be detrimental by inappropriately elevating [Ca^2+^] under basal glucose conditions following reduced or absent gap junction coupling.

We only included stochastic noise in the K_ATP_ channel, yet inclusion of noise from other channels will likely increase [Ca^2+^] further. The level of noise in a single β-cell is also not well characterized and depends on the number of channels. We estimated noise based on ~420 channels in a β-cell, which is consistent with reported channel populations [21,82]. The [Ca^2+^] predicted in normal islets in the absence of electrical coupling also matches experimental results [9,10]. However precisely examining noise characteristics in β-cells and their link to channel numbers will be important to accurately estimate [Ca^2+^] increases, especially in the presence of altered K_ATP_ kinetics.

### Conclusion

We applied computational and experimental approaches to examine the role of gap junction coupling in islet dysfunction caused by *KCNJ11* and *ABCC8* mutations. Gap junction electrical coupling strongly mediates the suppression of islet [Ca^2+^] and insulin secretion in the presence of ATP-insensitive K_ATP_ channels, and significant recovery in [Ca^2+^] can be achieved upon reduced electrical coupling. Following experimental validation of our computational model, we made firm predictions that such recovery could be achieved for many *KCNJ11* and *ABCC8* mutations that cause NDM. Increased [Ca^2+^] occurs through reducing the suppression of more excitable cells in the islet, allowing them to regain excitability. However stochastic noise also likely plays a role in elevating [Ca^2+^]. These results gain further insight into how the dysfunction to the islet of Langerhans can occur in disease, and suggests potential for therapeutic treatments for NDM where sulfonylureas are ineffective. Further the principles discovered that govern how connectivity of excitable and inexcitable β-cells dictate overall function in the islet may be applicable to other multicellular systems.

## Methods

### Ethics statement

All experiments were performed in compliance with the relevant laws and institutional guidelines, and were approved by the University of Colorado Institutional Biosafety Committee (IBC) and Institutional Animal Care and Use Committee (IACUC).

### Animal lines

The Kir6.2 subunit mutation with GFP tag (Rosa26-Kir6.2^[∆N30,K185Q]^); β-cell specific, inducible Cre (Pdx-Cre^ER^); and Connexin36 knockout (Cx36^-/-^) mouse models have been previously described [45,83,84]. Pdx-Cre^ER^ and Rosa26-Kir6.2^[∆N30,K185Q]^ mice were crossed with Cx36 knockout mice to yield all combinations of mice studied. Daily injections of tamoxifen (1–5 doses, 50 mg/kg body weight per dose) administered IP in 8–16 week old mice induced the variable Kir6.2^[∆N30,K185Q]^ expression. Mice lacking Pdx-Cre^ER^ or Rosa26- Kir6.2^[∆N30,K185Q]^ were used as 0% expression controls.

### Blood glucose and plasma insulin measurements

Blood glucose was measured daily after tamoxifen induction using a glucometer (Ascensia Contour, Bayer). Reported levels were averaged over days 27–29 after tamoxifen induction. Plasma insulin was measured at day 29 after tamoxifen induction from blood samples, centrifuged for 15 minutes at 13,900rpm, then assayed using mouse ultrasensitive insulin ELISA (Alpco).

### Islet isolation

Islets were isolated from mice under Ketamine/Xylazine anesthesia by collagenase injection through the pancreatic duct, and animals euthanized via exsanguination and cervical dislocation. Islets were handpicked after the pancreas was harvested and digested, and were maintained in RPMI medium at 11mM glucose plus 10% FBS, 100 U/ml penicillin, 100 μg/ml streptomycin, at 37°C under humidified 5% CO_2_ for 24–48 hours prior to study.

### Insulin secretion measurements

Islets (5/batch, duplicate) were incubated first at 2mM glucose in Krebs-Ringer Buffer (128.8mM NaCl, 5mM NaHCO_3_, 5.8mM KCl, 1.2mM KH_2_PO_4_, 2.5mM CaCl_2_, 1.2mM MgSO_4_, 10mM HEPES, 0.1% BSA, pH 7.4), and then for 60 minutes at 2mM or 20mM glucose. The medium was sampled for secretion, and islets sampled for content by lysing in 1% TritonX-100 and frozen overnight at -20C. Samples were assayed using mouse ultrasensitive ELISA.

### Calcium imaging

Isolated islets were loaded with 3μM Rhod-2 (Invitrogen), in imaging medium (125mM NaCl, 5.7mM KCl, 2.5mM CaCl_2_, 1.2mM MgCl_2_, 10mM Hepes, 2mM glucose, and 0.1% BSA, pH 7.4) for 45 minutes at room temperature, and were held in polymdimethylsiloxane PDMS microfluidic devices [85] maintained at 37°C. Rhod-2 fluorescence was imaged on a spinning disk confocal microscope (Marianas, 3I), excited at 561nm using an OPSL sapphire laser, with a 580-655nm band-pass filter for emission; or imaged on a confocal microscope (LSM780, Zeiss) excited at 561nm using a diode-pumped solid-state laser, with a 570-645nm band-pass selection for emission. A small sub-set of isolated islets were loaded with 4μM FuraRed (Invitrogen) for 90 minutes at room temperature, and imaged on a spinning disk confocal microscope, excited at 488nm using a diode-pumped solid-state laser, with a 580-655nm band-pass filter for emission. GFP fluorescence was excited at 488nm using a Ar+ laser line (LSM780) or diode-pumped solid-state laser (Marianas), with a 495-555nm band-pass selection for emission. Images were acquired 1/sec, 10 minutes after elevating glucose concentration (2-20mM). Microscope settings (integration time, scan time, gain, laser power) were constant for all images collected within the same day.

### Coupled β-cell electrical activity model

The model is modified from that described previously [27], and based on the Cha-Noma β-cell model [86] with cell-cell coupling and altered K_ATP_ channel function. Model code and associated files are included as supporting files. All model code was written in C or C++ and run on the University of Colorado JANUS supercomputer.

The membrane potential (*V*_*i*_) of each β-cell *i* is related to the total transmembrane current (*I*_*i*_), which is composed of individual currents described in [86]; using parameters in S3 Table:

$$-C\;V_{i}^{\prime }=I_{Cav}+I_{TRPM}+I_{SOC}+I_{bNSC}+I_{KDr}+I_{KCa(SK)}+I_{K(ATP)}+I_{NaK}+I_{NaCa}+I_{PMCA}$$ (1)

Gap junction coupling is modeled by assigning a coupling current between neighboring cells (*i*,*j*). A sphere packing algorithm was used to assemble cells within the cluster (mean number of cell-cell connections = 5.3) [27,87]

$$-C\;V_{i}^{\prime }=I_{i}+\sum_{i}g_{coup}^{i,j}(V_{i}-V_{j})$$ (2)

Heterogeneity in coupling was included by randomly assigning *g*^*i*,*j*^_*coup*_ according to a distribution from previously published data with SD/mean = 70% [47]. Endogenous heterogeneity was modeled by randomizing all parameters indicated in S3 Table between cells about a mean value according to a Gaussian distribution with SD/mean as indicated.

The K_ATP_ channel current was described as:

$$I_{K(ATP)}=g_{K(ATP)}∙p_{0K(ATP)}∙(V-V_{K})$$ (3)

where the open channel probability *po*_*K*(*ATP*)_ is given by:

$${po}_{K(ATP)}=\frac{.08(1+\frac{2[ADP]}{.01})+.89{\left(\frac{\left[ADP\right]}{.01}\right)}^{2}}{{\left(1+\frac{\left[ADP\right]}{.01}\right)}^{2}(1+\frac{.45[ADP]}{.026}+{\left(\frac{\left[ATP\right]}{.05}\right)}^{})}$$ (4)

### Modeling K_ATP_ channel mutations

Expression of Kir6.2^[ΔN30,K185Q]^ in rodent islets was modeled as previously and in accordance with experimental data [24,27], by modifying the open probability *po*_*K*(*ATP*)_ in a fraction (*P*_*mut*_) of cells, according to:

$$po_{K(ATP)[\Delta N30,K185Q]}=\gamma (po_{K(ATP)})+(1-\gamma )$$ (5)

where *γ* = 0.5 and *P*_*mut*_ increases with number of GFP^+^ Kir6.2^[ΔN30,K185Q]^ -expressing cells. Simulations for *P*_*mut*_ were run with glucose elevated to 20mM.

Diazoxide application was modeled as previously [27] by modifying the open probability *po*_*K*(*ATP*)_ in all cells according to:

$$po_{K(ATP)Diaz}=\alpha +(1-\alpha )*po_{K(ATP)}$$ (6)

where *α* is variable and increases with diazoxide treatment concentration. Simulations for *α* were run with glucose elevated to 11mM and *P*_*mut*_ = 100%.

*KCNJ11* and *ABCC8* mutations were modeled by modifying the open probability *po*_*K*(*ATP*)_ in all cells according to:

$${po}_{K(ATP)}=(1-\alpha )\frac{p_{o}^{\prime }*0.08(1+\frac{2[ADP]}{.01})+.89{\left(\frac{\left[ADP\right]}{.01}\right)}^{2}}{{\left(1+\frac{\left[ADP\right]}{.01}\right)}^{2}(1+\frac{.45[ADP]}{.026}+{\left(\frac{\left[ATP\right]}{k_{1/2}^{\prime }*0.05}\right)}^{H})}+\alpha$$ (7)

where ***k***^***’***^_***1/2***_ represents the relative increase in half maximal ATP concentration, and ***H*** is the Hill coefficient. Unless noted *H* = 1 and was not varied when modeling mutations in order to maintain consistency. ***p’***_***0***_ represents the relative increase/decrease in open channel conductance. ***α*** represents the fraction of current remaining at saturating ATP concentrations, equivalent to *α* in Eq 6; and was included if reported otherwise set to zero. To generate estimated *α* values (*α*_*est*_) for a mutation, data reporting the fractional K_ATP_ current remaining at 3mM ATP [57] was compared to the *po*_*K*(*ATP*)_ at 3mM ATP when including the reported ***k***^***’***^_***1/2***_. The difference between these values represents the estimated ATP-independent residual current (*α*_*est*_); where negative values were set to zero. Sources and parameter values for each mutation examined are summarized in S1 Table. All mutations were modeled from data reporting mixed mutant and wildtype subunit expression in heterologous expression systems. Simulations were run with glucose elevated to 11mM and *P*_*mu*t_ = 100%.

### Noise component of model

Stochastic noise was applied to the K_ATP_ current by including a time varying noise component, as previously reported [44]. Eq 3 was adjusted:

$$I_{KATP}=g_{kATP}∙[po_{k(ATP)}∙(1+S)]∙(V_{M}-V_{k})$$ (8)

where S represents the time varying noise component which fluctuates with mean≈0 and standard deviation≈0.049. The time varying noise component S was modeled as:

$$S\prime =\frac{-S}{\tau }-\left(\frac{S}{\tau }\right)+\xi$$ (9)

where τ = 500ms, and ξ represents a noise factor generated according to a random number sequence, such that S follows a normal distribution. The standard deviation of ξ was adjusted such that the standard deviation of S(t) (S_σ_ = 1/√N_K(ATP)_) was equivalent to ~420 channels per cell [44]. Similar noise was observed over different simulations (S9 Fig).

### Insulin secretion component of model

The general form of this component was adapted from a previously published insulin secretion model [58]. Insulin granules are designated in distinct pools, with rates of exchange leading up to a secretion event. The granule fusion step was modeled with [Ca^2+^] dependence to give a first phase release of ~20 granules/min per β-cell, and second phase rate of ~5 granules/min per β-cell [58,61]. Other rates were adjusted from those previously reported [58] to account for simplifications made to incorporate this component with our model. Specific rates, initial conditions and other parameters can be found in S4 Table.

$$Reserve\;Pool:\;RES^{\prime }=r_{res}RES-r_{\_res}RES$$ (10)

$$Docked\;Pool:\;DP^{\prime }=r_{-2}PP-r_{2}DP+r_{res}RES-r_{-res}RES$$ (11)

$$Primed\;Pool:\;PP^{\prime }=r_{-1}IRP-{(r}_{1}+r_{-2})∙PP+r_{2}DP$$ (12)

$$Immediate\;Release\;Pool:\;IRP^{\prime }=r_{1}PP-r_{-1}∙IRP-{fusion}_{I}∙IRP$$ (13)

$$Fusion\;Pool:\;FP^{\prime }=fusion_{I}∙IRP-u_{2}FP$$ (14)

$$Release\;Pool:\;RP^{\prime }=u_{2}FP-u_{3}RP$$ (15)

$$Granules\;Secreted\;per\;minute:\;Gran_{min}=u_{3}∙RP∙60$$ (16)

$$Total\;granules\;secreted:\;Total_{gran}=\int u_{3}∙RP$$ (17)

$$Fusion\;[{Ca}^{2+}]\;dependence:\;fusion_{I}=fusion_{Max}∙\frac{Ca_{i}^{nFuse}}{Ca_{i}^{nFuse}+K_{I}^{nFuse}}$$ (18)

### Calcium imaging data analysis

Custom MATLAB scripts were used to analyze all images acquired from calcium imaging [31]. First, images were smoothed using a 5x5 average filter. To calculate the fraction of cells showing elevated [Ca^2+^], a quiescent reference cell was selected manually from an area where no significant intensity fluctuations occurred over the duration of the experiment. The variance for each pixel time-course of the image was calculated to examine which pixels displayed the greatest fluctuations in time. The variance of the manually selected quiescent reference cell was used to generate a threshold to compare intensity fluctuations of all other pixels: any pixels having a time-course variance > 2 standard deviations above that of the quiescent reference cell were counted as ‘active’. Time courses with significant motion artifacts were excluded from analysis, and photobleaching was handled by applying a linear fit. The percentage of cells active is calculated based on the number of pixels calculated to be ‘active’, normalized to the number of pixels in the area of the islet. To calculate GFP^+^ regions, the mean fluorescence intensity was calculated in GFP^-^ control islets cells and used to generate an intensity threshold: any pixel having a GFP intensity greater than this threshold was considered GFP^+^. GFP+ area was expressed as a % of the total islet area. In islets where lack of nuclear GFP was observed, nuclear areas were manually included in the GFP^+^ area. For each level of Cx36 expression, the average activity over all control islets was used to normalize the maximum activity of simulation fits.

### Simulation data analysis

All analysis was performed with custom MATLAB routines on the output for each simulation. For all time courses, the first ~200 time points were excluded. To calculate the fraction of cells showing elevated [Ca^2+^], a threshold of 0.165μM was applied, and cells showing [Ca^2+^] fluctuations that exceeded this threshold were considered active. The [Ca^2+^] duty cycle for each cell was represented by the fraction of time the [Ca^2+^] time course exceeded this threshold. The duty cycle was reported as the mean duty cycle over all cells in the islet, where a silent cell has a duty cycle of 0. The same analysis methods were used when noise was added to the model.

The total number of granules secreted was calculated according to Eq 17 over 30 minutes of the simulation time course. First phase secretion was calculated according to Eq 17 over the first 5 minutes after insulin elevated. Second phase secretion was calculated from t = 5 min to t = 30 minutes after insulin elevated. All values were normalized to those of control islet simulations ran using the same heterogeneity and coupling distributions.

### Statistical analysis

Student’s t-test was utilized to test for significant differences between simulation results; [Ca^2+^] imaging results; or insulin secretion, plasma insulin and blood glucose results. Linear regression between [Ca^2+^] imaging results, insulin secretion, plasma insulin and blood glucose against % GFP was grouped across Cx36^+/+^,Cx36^+/-^,Cx36^-/-^ conditions. All statistical analysis was performed in Prism (Graphpad).

## Supporting Information

S1 Table Table of parameters used to model alerted ATP inhibition kinetics in mutant K_ATP_ channels. nR: not reported.
(PDF)

S2 Table Summary of the simulated effects of reduced electrical coupling, stochastic channel noise and sulfonylurea treatment on each mutation examined. A ‘Yes’ for calcium disruption refers to a decrease in Fraction active of <50%. For different Recovery categories, ‘+’refers to >0%-10%; ‘++’ refers to 10–50%; ‘+++’ refers to >50%.
(PDF)

S3 Table Table of parameters used in electrophysiology model, where all nomenclature is consistent with the published single cell β-cell Cha-Noma model, see [86]. *Heterogeneity is based on Gaussian variability about the given value with standard deviation indicated as percentage of the given value.
(PDF)

S4 Table Table of parameters used in insulin secretion component of model, see [58]. (PDF)

S1 Fig Individual data for physiological measurements. **A).** Scatter plots of (from top to bottom) insulin secretion, plasma insulin, blood glucose and fraction of cells showing significant [Ca^2+^] elevations, verses % GFP expression (indicating % Kir6.2^[ΔN30,K185Q]^ expression), for islets isolated from Cx36^+/+^ mice or measured from Cx36^+/+^ mice. **B).** As in A for Cx36^+/-^ mice. **C).** As in A for Cx36^-/-^ mice. Linear regression ± 95% CI indicated by grey lines.
(PDF)

S2 Fig Phase transitions in islet activity as shown by the activity in the coupled islet system as a function of the activity in the uncoupled islet. **A).** Islet activity for simulations at the indicated *g*_*coup*_, as represented by the fraction of cells showing significant [Ca^2+^] elevations, plotted against intrinsic cellular activity, as indicated by fraction of cells showing significant [Ca^2+^] elevations for simulations at *g*_*coup*_ = 0pS. Displayed are results from simulations with increasing *k*^*’*^_*1/2*_ at 11mM glucose, *P*_*mu*t_ = 1. **B).** As in A for results from simulations with increasing *α* at 11mM glucose, *P*_*mu*t_ = 1. **C).** As in A for results from simulations with increasing *P*_*mut*_ at 20mM glucose.
(PDF)

S3 Fig Complete results from simulations of Kir6.2 and SUR1 mutations for reported *α*. **A).** Fraction of cells showing significant [Ca^2+^] elevations for simulations that include mutant K_ATP_ channel activity, for *g*_*coup*_ = 120pS (black) and *g*_*coup*_ = 0pS (red). Simulations include all characterized mutations indicated where reported *k*^*’*^_*1/2*_ changes and reported *α* (if any) are accounted for. **B).** Mean [Ca^2+^] duty cycle for simulations that include mutant K_ATP_ channel activity as in A. All simulations were run at 11mM glucose, *P*_*mu*t_ = 1. Results are arranged in order of clinical severity, with the clinical classification indicated: T2D- (Type2 Diabetes; TNDM- Transient Neonatal Diabetes Mellitus; PNDM- Permanent Neonatal Diabetes Mellitus; DEND- PNDM with Developmental Delay and Neurological features, including iDEND. * indicates mutations where sulfonylurea therapy is reported to be ineffective.
(PDF)

S4 Fig Complete results from simulations of Kir6.2 and SUR1 mutations with inclusion of estimated *α*. **A).** Fraction of cells showing significant [Ca^2+^] elevations for simulations that include mutant K_ATP_ channel activity, for *g*_*coup*_ = 120pS (black) and *g*_*coup*_ = 0pS (red). Simulations include all characterized mutations indicated where reported *k*^*’*^_*1/2*_ changes and estimated *α* (see S1 Table) are accounted for. **B).** Mean [Ca^2+^] duty cycle for simulations that include mutant K_ATP_ channel activity as in A. All simulations were run at 11mM glucose, *P*_*mu*t_ = 1. Results are arranged in order of clinical severity, with the clinical classification indicated: T2D- (Type2 Diabetes; TNDM- Transient Neonatal Diabetes Mellitus; PNDM- Permanent Neonatal Diabetes Mellitus; DEND- PNDM with Developmental Delay and Neurological features, including iDEND. * indicates mutations where sulfonylurea therapy is reported to be ineffective.
(PDF)

S5 Fig Simulated effect of stochastic channel noise on [Ca^2+^] for selected Kir6.2 and SUR1 mutations with inclusion of estimated *α*. **A).** Fraction of cells showing significant [Ca^2+^] elevations for simulations that include mutant K_ATP_ channel activity, with stochastic noise, for left: *g*_*coup*_ = 120pS (black) and *g*_*coup*_ = 0pS (red), and right: *g*_*coup*_ = 120pS (black) and *g*_*coup*_ = 10pS (green). Simulations include the characterized mutations indicated, where reported *k*^*’*^_*1/2*_ changes and estimated *α* (see S2 Table) are accounted for. **B).** Mean [Ca^2+^] duty cycle for simulations that include mutant K_ATP_ channel activity, with stochastic noise, as in A. All simulations were run at 11mM glucose, *P*_*mu*t_ = 1. Results are arranged in order of clinical severity. * indicates mutations where sulfonylurea therapy is reported to be ineffective. Data is presented as mean±s.e.m. for n = 3 simulations with different random number seeds.
(PDF)

S6 Fig Verification of insulin secretion dynamics in simulated single β-cell. **A).** Insulin secretion following step increase in [Ca^2+^] at t = 5 min, showing biphasic response. **B).** Insulin secretion following step increase in glucose at t = 5 min, showing biphasic response.
(PDF)

S7 Fig Recovery of insulin secretion by first phase and second phase. **A).** Time-averaged insulin secretion over first phase of secretion (t = 0-5min.) for simulations that include mutant K_ATP_ channel activity for *g*_*coup*_ = 120pS and *g*_*coup*_ = 0pS, with stochastic channel noise. Simulations include the characterized mutations indicated where reported *k*^*’*^_*1/2*_ changes and reported *α* (if any) are accounted for. **B).** Time-averaged insulin secretion over second phase of secretion (t = 5-30min.) for simulation conditions in A. **C).** Time-course of the population of insulin granule pools averaged over a representative simulated WT islet with *g*_*coup*_ = 120pS and stochastic noise included, corresponding to data presented in Fig 8D. **D).** Time-course of the population of different insulin granule pools, as in C, for a simulated islet that includes I296L mutation for *g*_*coup*_ = 120pS (black) and *g*_*coup*_ = 0pS (red), corresponding to data presented in Fig 8D. All simulations were run at 11mM glucose, *P*_*mu*t_ = 1. Data in A,B is presented as mean for n = 2 simulations.
(PDF)

S8 Fig [Ca^2+^] and insulin time-courses of sulfonylurea action. **A).** Representative [Ca^2+^] time courses for simulations upon reduced *p*^*’*^_*o*_ for *g*_*coup*_ = 0pS with stochastic channel noise. **B).** As in A for simulations that include mutant K_ATP_ channel activity. **C).** Representative time-courses of insulin secretion averaged across the simulated islet for simulations in A, with normal and reduced *p*^*’*^_*o*_. **D).** As in C for simulations in B, with normal and reduced *p*^*’*^_*o*_. All simulations were run at 11mM glucose, *P*_*mu*t_ = 1.
(PDF)

S9 Fig Characterizing the effect of stochastic noise at low glucose. **A).** Fraction of cells showing significant [Ca^2+^] elevations for simulations at 5.5mM glucose with g_coup_ = 0pS, without noise and for 10 different simulations with stochastic channel noise. **B).** [Ca^2+^] duty cycle for simulations in A.
(PDF)

S1 File Source code files used to simulate the islet with H46Y mutation, that generates data presented in Fig 8G and S5 Fig. The ‘read_me.doc’ file included explains how the source code files were used.
(ZIP)
