# Supplementary material for: Decreases in Gap Junction Coupling Recovers Ca2+ and Insulin Secretion in Neonatal Diabetes Mellitus, Dependent on Beta Cell Heterogeneity and Noise
Source: PLoS Comput Biol. 2016 Sep 28;12(9):e1005116. doi: 10.1371/journal.pcbi.1005116 (PMC5040430; doi:10.1371/journal.pcbi.1005116)
Supplement: S1 Table — nR: not reported. (PDF) [file pcbi.1005116.s001.pdf]

KIR6.2 MUTATIONS									
**Mutation**	**$K'_{1/2}$**	**P'_o**	**Hill Coefficient WT(Mutant)**	**α Reported**	**p_{KATP} (3mM ATP)**	**p_{KATP} (3mM) Modeled**	**α Estimated**	**Phenotype**	**Reference**
WT	1.000	1.00	1.000	0.000	0.000	0.016	n/a	none	
E23K	1.333	nR	1.3(1.3)	nR	nR	0.022	0.000	T2D	Villareal et al. 2009
E229K	5.460	1.78	.93(0.95)	nR	0.030	0.083	0.000	TNDM	Girard et al. 2006
V252A	2.730	nR	.93(0.53)	nR	0.085	0.044	0.041	TNDM	Girard et al. 2006
G53S	1.851	nR	nR	nR	0.040	0.030	0.010	TNDM	Gloyn et al. 2005
G53R	1.522	nR	nR	nR	0.053	0.025	0.028	TNDM	Gloyn et al. 2005
I182V	1.612	nR	nR	nR	0.050	0.026	0.024	TNDM	Gloyn et al. 2005
R201H	1.500	nR	1.1(1.6)	nR	0.040	0.024	0.016	PNDM	Gloyn et al. 2004
R50Q	2.870	nR	.97(1.37)	nR	0.044	0.046	0.000	PNDM	Shimomura et al. 2006
H46Y	6.200	nR	.93(0.68)	nR	0.066	0.094	0.000	PNDM	Girard et al. 2006
E292G	6.400	nR	.93(0.8)	nR	0.067	0.096	0.000	PNDM	Girard et al. 2006
N48D	11.667	nR	.93(.56)	nR	0.153	0.163	0.000	PNDM	Proks et al. 2004
E227K	14.200	nR	.93(.84)	nR	0.093	0.191	0.000	PNDM	Girard et al. 2006
F35L	2.848	nR	1.1(1.3)	nR	0.040	0.045	0.000	PNDM	Proks et al. 2006
*L164P	10.417	nR	1.3(1.1)	nR	0.336	0.148	0.188	PNDM	Tammaro et al. 2008
F333I	2.151	nR	1.39(1.01)	nR	0.090	0.035	0.055	PNDM	Tammaro et al. 2005
R50P	3.130	nR	.97(.88)	nR	0.327	0.050	0.277	PNDM	Shimomura et al. 2006
F35V	2.580	nR	1.1(1.2)	nR	0.075	0.041	0.034	PNDM	Proks et al. 2006
R201C	1.500	nR	nR	nR	0.148	0.024	0.124	PNDM/DEND	Proks et al. 2006
Y330C	1.858	nR	1.39(1.08)	nR	0.025	0.030	0.000	PNDM/DEND	Tammaro et al. 2005
V59M	2.420	nR	1.07(0.94)	0.100	0.128	0.039	0.089	iDEND	Proks et al. 2005
I167L	13.000	nR	.97(.78)	nR	0.273	0.178	0.095	iDEND	Shimomura et al. 2007
*Q52R	3.280	1.32	1.9(1.2)	nR	0.260	0.052	0.208	DEND	Proks et al 2004
*I296L	6.770	nR	1.3(1.3)	nR	0.310	0.101	0.209	DEND	Koster et al. 2005
*V59G	3.710	1.32	1.18(1.18)	nR	0.398	0.058	0.340	DEND	Proks et al. 2005
*T293N	6.167	nR	nR	nR	0.300	0.093	0.207	DEND	Shimomura et al. 2009
F60Y	9.300	nR	nR	nR	0.200	0.134	0.066	DEND	Mannikko et al. 2010
V64L	1.300	nR	nR	nR	0.010	0.021	0.000	DEND	Mannikko et al. 2010
*G334D	2.990	nR	nR	0.100	0.390	0.047	0.343	DEND	Masia et al. 2007
SUR1 MUTATIONS									
Y356C	3.125	nR	nR	nR	0.010	0.050	0.000	T2D	Tarasov et al. 2008
hetT229I	1.133	nR	1.11(1.11)	nR	0.012	0.019	0.000	TNDM	Ellard et al. 2007
hetL582V	2.125	nR	nR	nR	0.090	0.034	0.056	TNDM	Tarasov et al. 2008
hetF132L	6.375	nR	.93(0.75)	nR	0.060	0.096	0.000	DEND	Proks et al. 2006

Table S1
