# Supplementary material for: Decreases in Gap Junction Coupling Recovers Ca2+ and Insulin Secretion in Neonatal Diabetes Mellitus, Dependent on Beta Cell Heterogeneity and Noise
Source: PLoS Comput Biol. 2016 Sep 28;12(9):e1005116. doi: 10.1371/journal.pcbi.1005116 (PMC5040430; doi:10.1371/journal.pcbi.1005116)
Supplement: S2 Table — A ‘Yes’ for calcium disruption refers to a decrease in Fraction active of <50%. For different Recovery categories, ‘+’refers to >0%-10%; ‘++’ refers to 10–50%; ‘+++’ refers to >50%. (PDF) [file pcbi.1005116.s002.pdf]

Mutation	Phenotype	Calcium Disrupted (g_{coup} =120pS)		g_{coup} = 0pS Recovery		g_{coup} = 0pS with Noise Recovery		Simulated SU Recovery (0pS)
		$K'_{1/2}$	$K'_{1/2}$+ α	$K'_{1/2}$	$K'_{1/2}$ + α	$K'_{1/2}$	$K'_{1/2}$ + α	$K'_{1/2}$ + α
WT	none							
E23K	T2D	No	No	-	-	-	-	
E229K	TNDM	Yes	Yes	++	++	+++	+++	
V252A	TNDM	No	Yes	-	-	+	+	++
G53S	TNDM	No	No	-	-	-	-	
G53R	TNDM	No	Yes	-	++	-	+++	
I182V	TNDM	No	Yes	-	++	-	+++	
R201H	PNDM	No	No	-	-	-	-	
R50Q	PNDM	No	No	-	-	-	-	
H46Y	PNDM	Yes	Yes	++	++	+++	+++	
E292G	PNDM	Yes	Yes	+	+	++	+++	
N48D	PNDM	Yes	Yes	-	-	-	-	
E227K	PNDM	Yes	Yes	-	-	-	-	
F35L	PNDM	No	No	-	-	-	-	
*L164P	PNDM	Yes	Yes	-	-	-	-	
F333I	PNDM	No	Yes	-	-	-	+	+
R50P	PNDM	No	Yes	-	-	-	-	
F35V	PNDM	No	Yes	-	+	-	++	++
R201C	PNDM/DEND	No	Yes	-	-	-	-	
Y330C	PNDM/DEND	No	No	-	-	-	-	
V59M	iDEND	Yes	Yes	-	-	-	-	
I167L	iDEND	Yes	Yes	-	-	-	-	
*Q52R	DEND	No	Yes	-	-	-	-	
*I296L	DEND	Yes	Yes	+	-	++	-	
*V59G	DEND	No	Yes	++	-	-	-	
*T293N	DEND	Yes	Yes	++	-	++	-	
F60Y	DEND	Yes	Yes	-	-	-		
V64L	DEND	No	No	-	-	-		
*G334D	DEND	No	Yes	-	-	-	-	
SUR1 Mutations								
Y356C	T2D	No	No	-	-	-	-	
L582V	TNDM	No	Yes	-	-	-	+	+
T229I	TNDM	No	No	-	-	-	-	
F132L	DEND	Yes	Yes	+	+	+++	+++	
**$K'_{1/2}$= $K'_{1/2}$ Reported<br>α = α Estimated**								

+ : 0-10% increase
++: 10-50% increase
+++: >50% increase

Table S2
