# Supplementary material for: Decreases in Gap Junction Coupling Recovers Ca2+ and Insulin Secretion in Neonatal Diabetes Mellitus, Dependent on Beta Cell Heterogeneity and Noise
Source: PLoS Comput Biol. 2016 Sep 28;12(9):e1005116. doi: 10.1371/journal.pcbi.1005116 (PMC5040430; doi:10.1371/journal.pcbi.1005116)
Supplement: S3 Table — *Heterogeneity is based on Gaussian variability about the given value with standard deviation indicated as percentage of the given value. (PDF) [file pcbi.1005116.s003.pdf]

Independent Variable	Description	Value
$[Na^+]_o$	Extracellular Composition	140mM
$[K^+]_o$	Extracellular Composition	5.4mM
$[Ca^{2+}]_o$	Extracellular Composition	2.6mM
C_m	Cell Capacitance	6.158pF
vol_i	Cell Cytosol Volume	764fl
vol_{ER}	Endoplasmic Reticulum Volume	280fl
f_i	Cytosolic Ca^{2+} Buffer Strength	0.01
f_{ER}	ER Ca^{2+} Buffer Strength	0.025
P_{CaV}	Converting factor for I_{CaV}	48.9 pA mM^{-1}
P_{KDr}	Converting factor for I_{KDr}	2.1 pA mM^{-1}
$G_{KCa(BK)}$	Conductance of $I_{kca(BK)}$	2.13 pA mV^{-1} (10%)*
$P_{KCa(SK)}$	Converting factor of $I_{KCa(SK)}$	0.2 pA mM^{-1}
P_{bNSC}	Converting factor of I_{bNSC}	0.00396 pA mM^{-1}
P_{SOC}	Converting factor of I_{SOC}	0.00764 pA mM^{-1}
$K_{0.SER}$	Half Activation Conc. Of Ca^{2+} in ER	0.003mM
$G_{K(ATP)}$	Max conductance of I_{KATP}	2.31 pA mV^{-1} (25%)*
P_{NaK}	Max amplitude of I_{NaK}	350 Pa ms
P_{NaCa}	Max amplitude of I_{NaCa}	204pA (10%)*
P_{PMCA}	Max amplitude of I_{PMCA}	1.56pA
P_{SERCA}	Max pump rate of Ca^{2+} into ER	0.096fl ms^{-1} (10%)*
P_{rel}	Converting factor for ER Ca^{2+} release	0.46fl ms^{-1} (10%)*
k_{glc}	Rate constant for glycolysis	0.000126 ms^{-1} (10%)*
$K_{\beta ox}$	Rate constant of β-oxidation	0.0000063 ms^{-1} (10%)*
Po_p	Max rate of ATP production	0.0005 ms^{-1} (10%)*
$[ATP_{tot}]$	Total ATP species	4mM (10%)*
k_{ATP}	Rate const of Ca^{2+} and ind. Ca^{2+} consumption	0.000062 ms^{-1}
$k_{ATP,Ca}$	Rate const of Ca^{2+} and dep. ATP consumption	0.187 mM-1 ms^{-1}
$K_{ADP,f}$	Rate constant of ADPf to ADPb	0.0002 ms^{-1}
$k_{ADP,b}$	Rate constant of ADPb to ADPf	0.00002 ms^{-1}

Table S3
