# Supplementary material for: Decreases in Gap Junction Coupling Recovers Ca2+ and Insulin Secretion in Neonatal Diabetes Mellitus, Dependent on Beta Cell Heterogeneity and Noise
Source: PLoS Comput Biol. 2016 Sep 28;12(9):e1005116. doi: 10.1371/journal.pcbi.1005116 (PMC5040430; doi:10.1371/journal.pcbi.1005116)
Supplement: S4 Table — (PDF) [file pcbi.1005116.s004.pdf]

Independent Variable	Description	Value
Fusion Max	Maximum granules fused with membrane	.030 ms^{-1}
N	Hill Coefficient	4
K_I	Half Maximal Ca_i for fusion	0.0022 mM
r_1	Rate from PP to IRP	0.02 ms^{-1}
r_{-1}	Rate from IRP to PP	0.025 ms^{-1}
r_2	Rate from DP to PP	0.00012 ms^{-1}
r_{-2}	Rate from PP to DP	0.0012 ms^{-1}
R_{res}	Rate from DP to RES	0.00005 ms^{-1}
R_{-res}	Rate from RES to DP	0.00004 ms^{-1}
u_2	Rate from FP to RP	0.003 ms^{-1}
u_3	Rate of Secretion from RP	0.00004 ms^{-1}
IRP(0)	I.C. for IRP	7.69
PP(0)	I.C. for PP	38
DP(0)	I.C. for DP	297
RES(0)	I.C. for RES	900
FP(0)	I.C. for FP	$1*10^{-7}$
RP(0)	I.C. for RP	$1*10^{-5}$
Cap(0)	I.C. for Membrane Capacitance	0

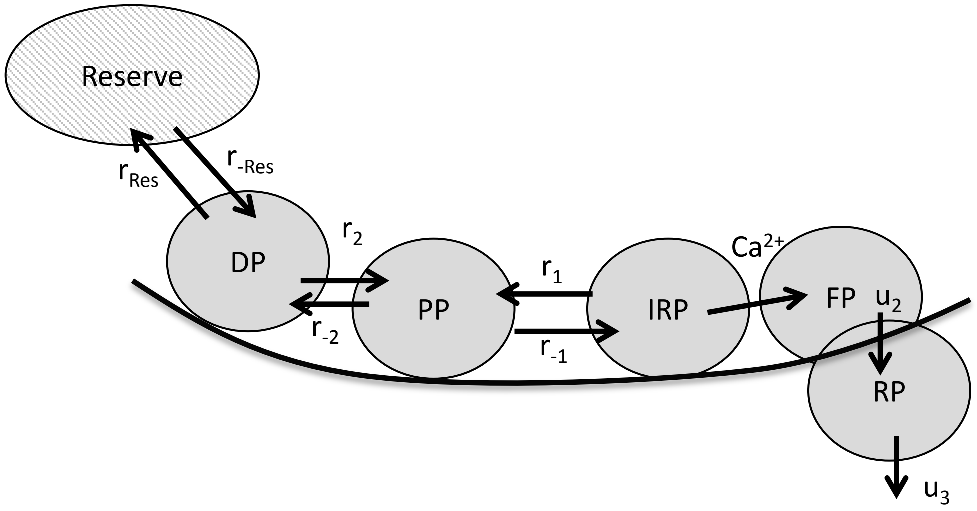

Table S4
