# Supplementary figures and images for: Decreases in Gap Junction Coupling Recovers Ca2+ and Insulin Secretion in Neonatal Diabetes Mellitus, Dependent on Beta Cell Heterogeneity and Noise
Source: PLoS Comput Biol. 2016 Sep 28;12(9):e1005116. doi: 10.1371/journal.pcbi.1005116 (PMC5040430; doi:10.1371/journal.pcbi.1005116)

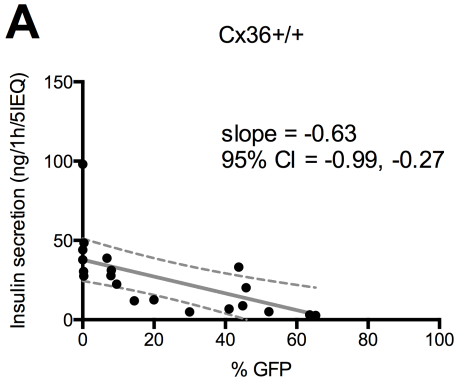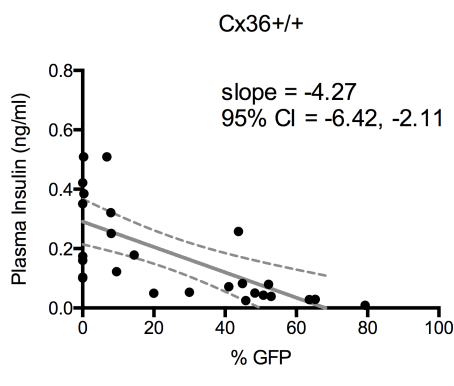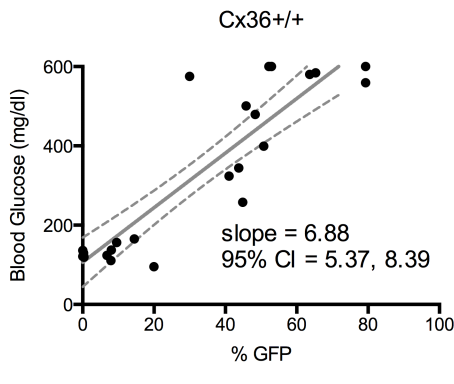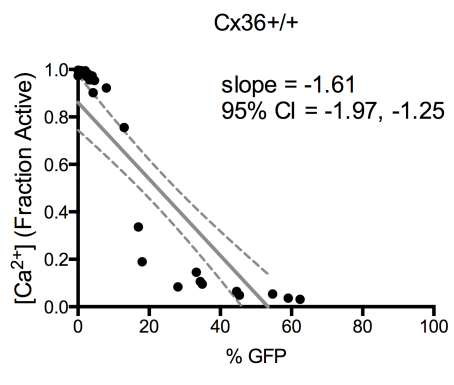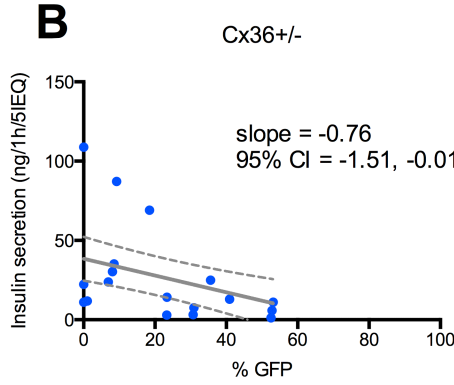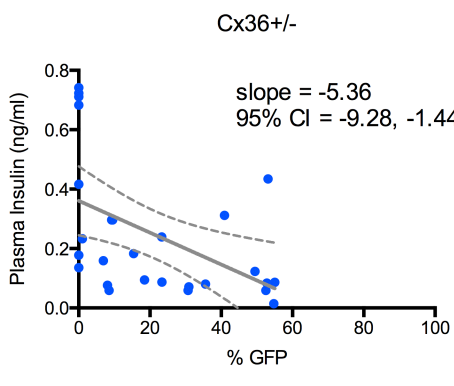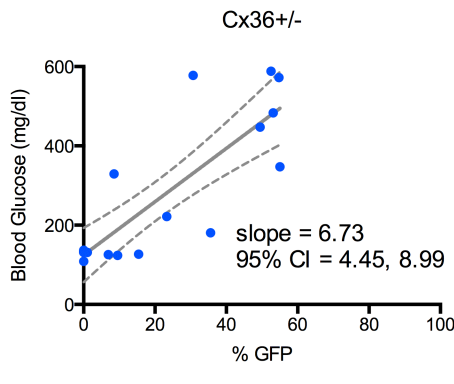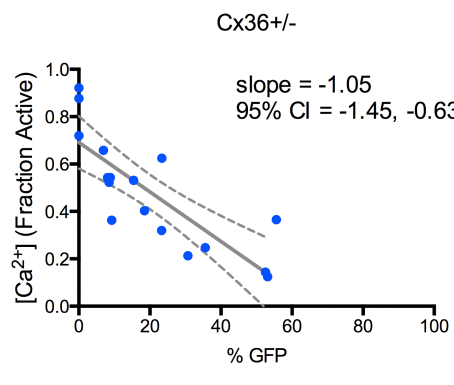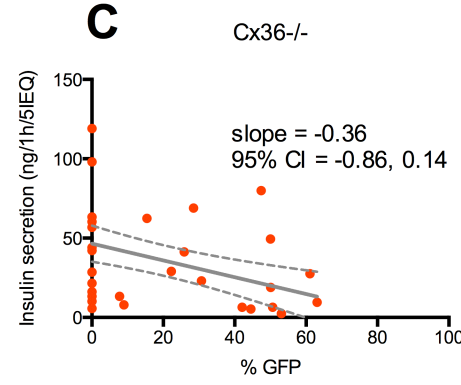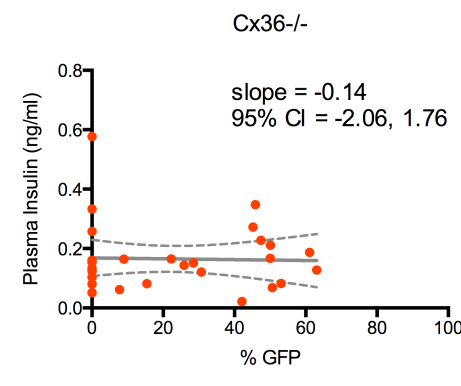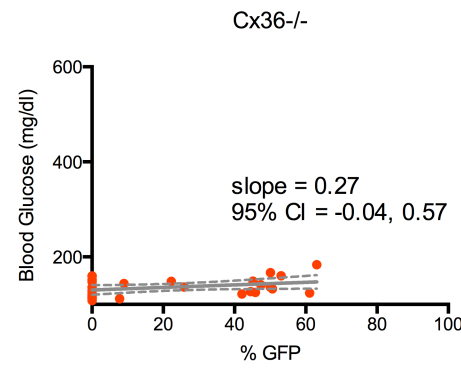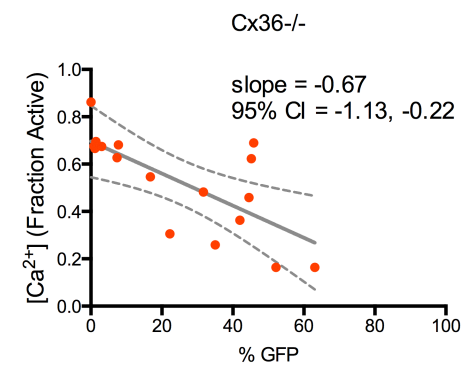

Figure S1

Supplement: S1 Fig — A). Scatter plots of (from top to bottom) insulin secretion, plasma insulin, blood glucose and fraction of cells showing significant [Ca2+] elevations, verses % GFP expression (indicating % Kir6.2[ΔN30,K185Q] expression), for islets isolated from Cx36+/+ mice or measured from Cx36+/+ mice. B). As in A for Cx36+/- mice. C). As in A for Cx36-/- mice. Linear regression ± 95% CI indicated by grey lines. (PDF) [file pcbi.1005116.s005.pdf]

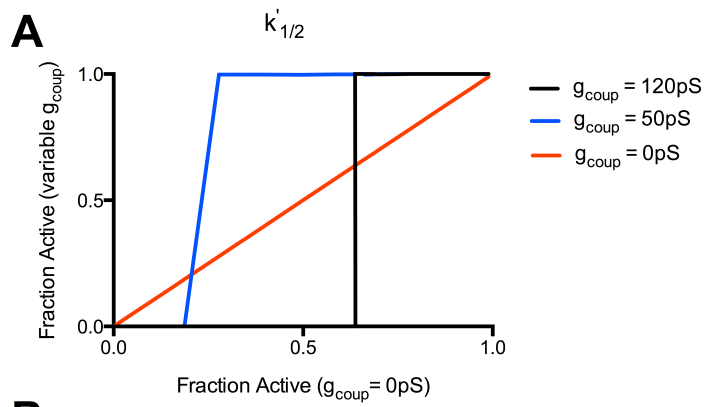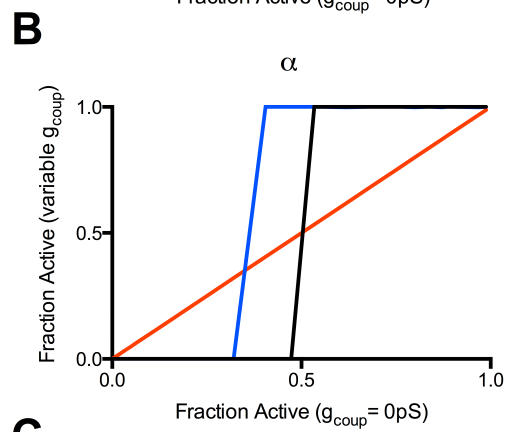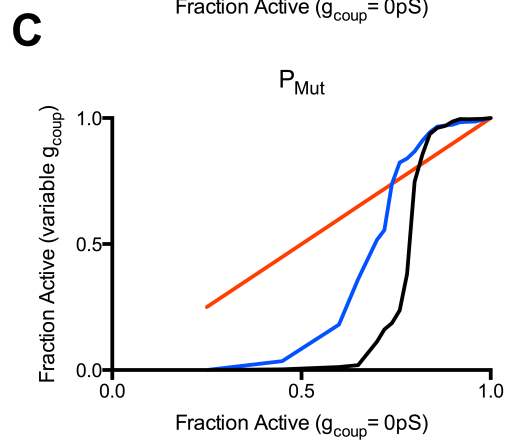

Figure S2

Supplement: S2 Fig — A). Islet activity for simulations at the indicated gcoup, as represented by the fraction of cells showing significant [Ca2+] elevations, plotted against intrinsic cellular activity, as indicated by fraction of cells showing significant [Ca2+] elevations for simulations at gcoup = 0pS. Displayed are results from simulations with increasing k’1/2 at 11mM glucose, Pmut = 1. B). As in A for results from simulations with increasing α at 11mM glucose, Pmut = 1. C). As in A for results from simulations with increasing Pmut at 20mM glucose. (PDF) [file pcbi.1005116.s006.pdf]

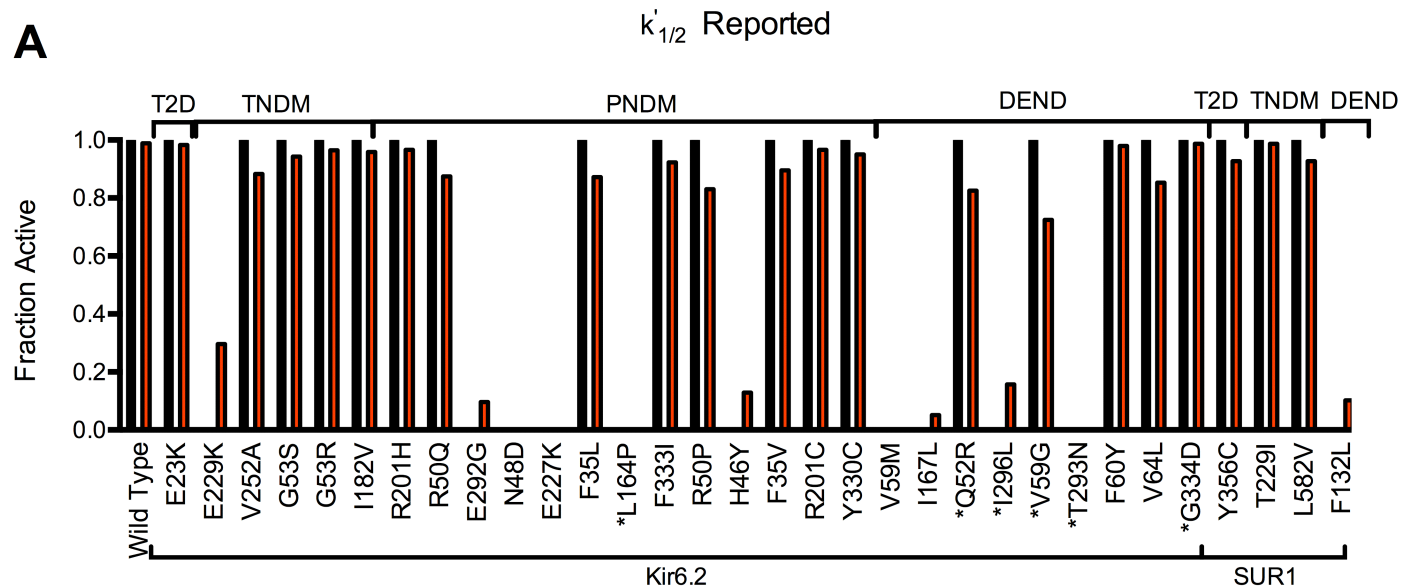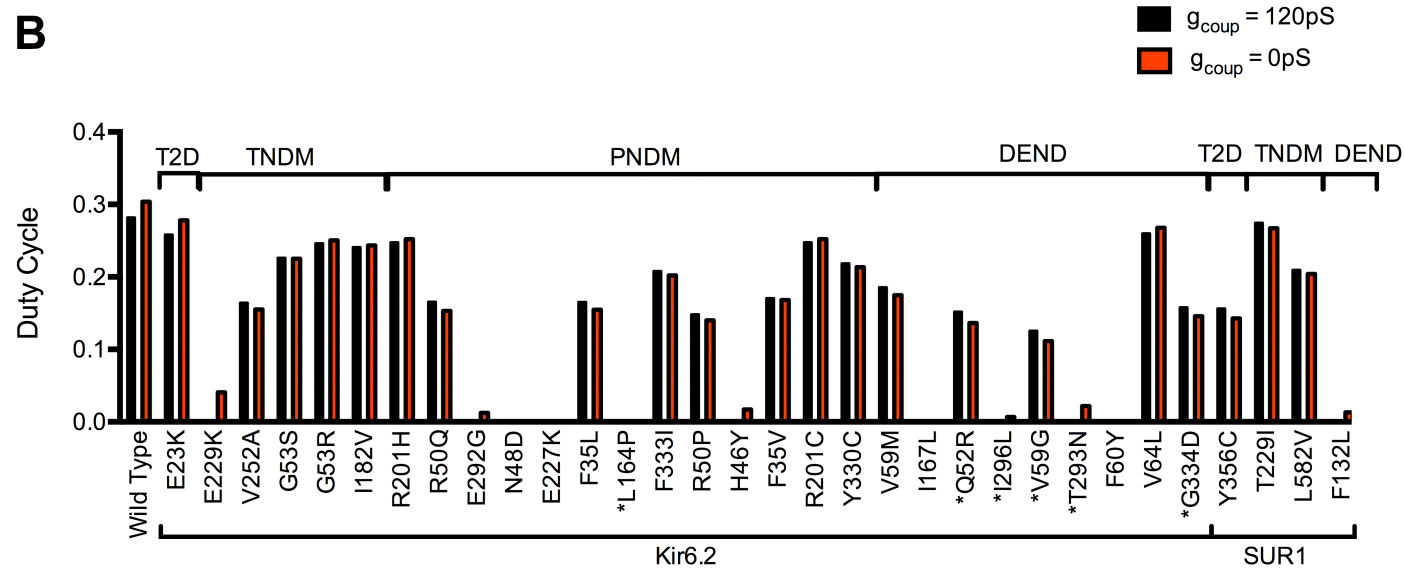

Figure S3

Supplement: S3 Fig — A). Fraction of cells showing significant [Ca2+] elevations for simulations that include mutant KATP channel activity, for gcoup = 120pS (black) and gcoup = 0pS (red). Simulations include all characterized mutations indicated where reported k’1/2 changes and reported α (if any) are accounted for. B). Mean [Ca2+] duty cycle for simulations that include mutant KATP channel activity as in A. All simulations were run at 11mM glucose, Pmut = 1. Results are arranged in order of clinical severity, with the clinical classification indicated: T2D- (Type2 Diabetes; TNDM- Transient Neonatal Diabetes Mellitus; PNDM- Permanent Neonatal Diabetes Mellitus; DEND- PNDM with Developmental Delay and Neurological features, including iDEND. * indicates mutations where sulfonylurea therapy is reported to be ineffective. (PDF) [file pcbi.1005116.s007.pdf]

**A** $k'_{1/2}$  Reported,  $\alpha$  Estimated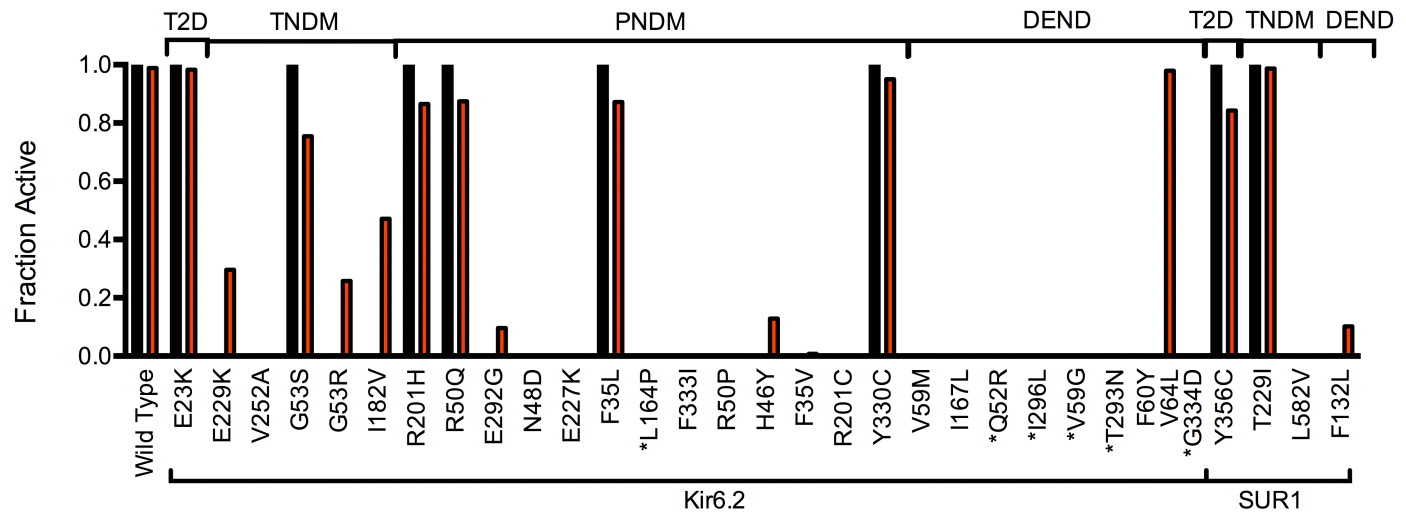**B**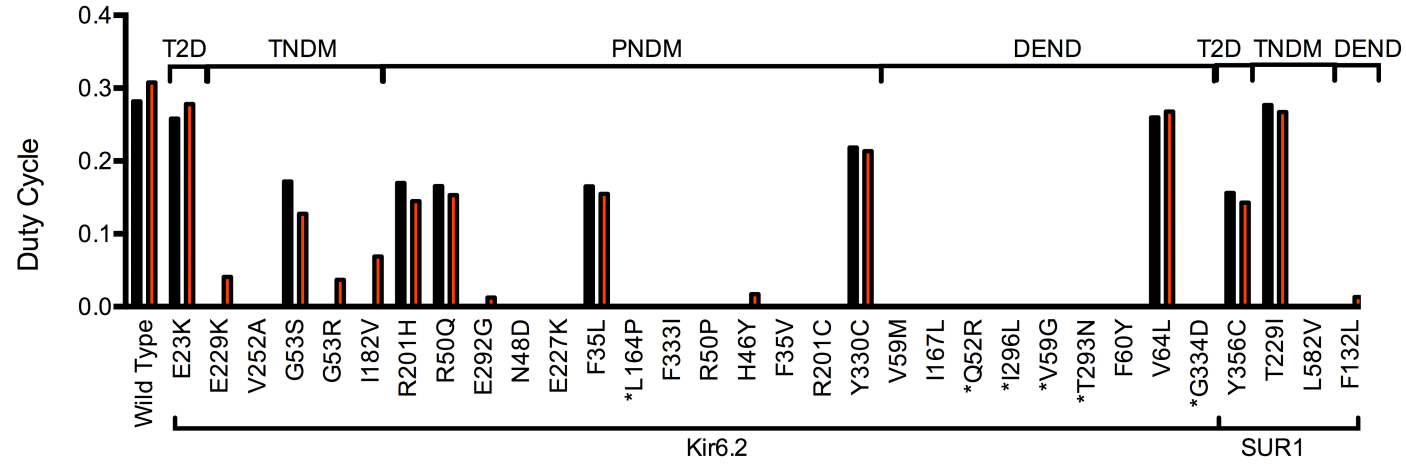

Figure S4

Supplement: S4 Fig — A). Fraction of cells showing significant [Ca2+] elevations for simulations that include mutant KATP channel activity, for gcoup = 120pS (black) and gcoup = 0pS (red). Simulations include all characterized mutations indicated where reported k’1/2 changes and estimated α (see S1 Table) are accounted for. B). Mean [Ca2+] duty cycle for simulations that include mutant KATP channel activity as in A. All simulations were run at 11mM glucose, Pmut = 1. Results are arranged in order of clinical severity, with the clinical classification indicated: T2D- (Type2 Diabetes; TNDM- Transient Neonatal Diabetes Mellitus; PNDM- Permanent Neonatal Diabetes Mellitus; DEND- PNDM with Developmental Delay and Neurological features, including iDEND. * indicates mutations where sulfonylurea therapy is reported to be ineffective. (PDF) [file pcbi.1005116.s008.pdf]

**A**
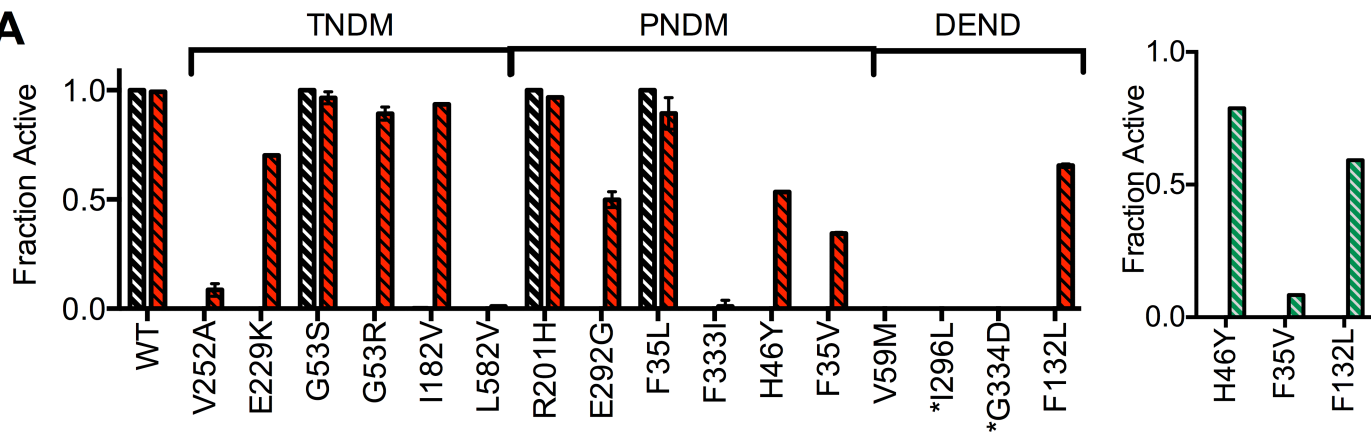
**B**
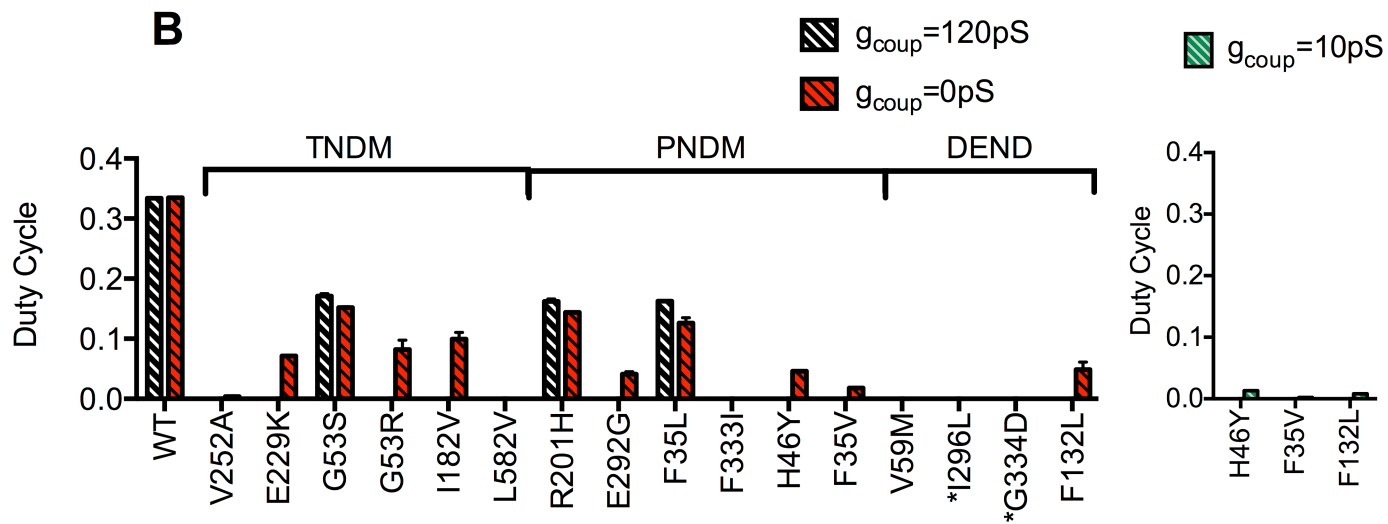

Figure S5

Supplement: S5 Fig — A). Fraction of cells showing significant [Ca2+] elevations for simulations that include mutant KATP channel activity, with stochastic noise, for left: gcoup = 120pS (black) and gcoup = 0pS (red), and right: gcoup = 120pS (black) and gcoup = 10pS (green). Simulations include the characterized mutations indicated, where reported k’1/2 changes and estimated α (see S2 Table) are accounted for. B). Mean [Ca2+] duty cycle for simulations that include mutant KATP channel activity, with stochastic noise, as in A. All simulations were run at 11mM glucose, Pmut = 1. Results are arranged in order of clinical severity. * indicates mutations where sulfonylurea therapy is reported to be ineffective. Data is presented as mean±s.e.m. for n = 3 simulations with different random number seeds. (PDF) [file pcbi.1005116.s009.pdf]

**A**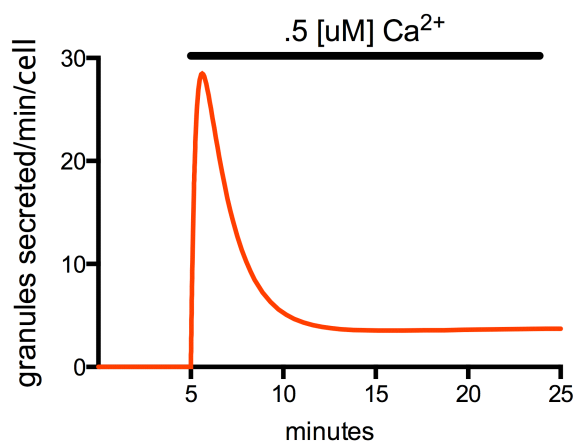**B**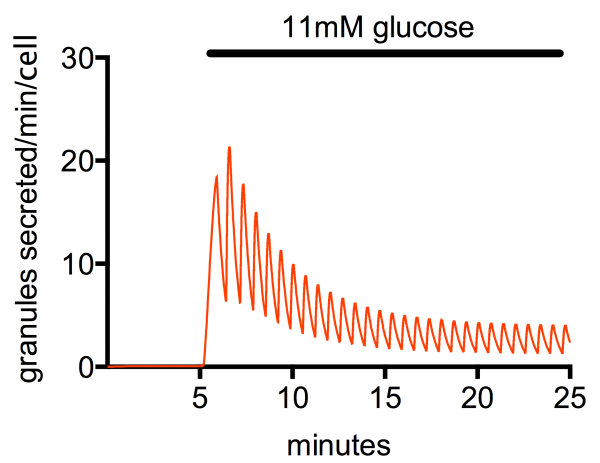

Figure S6

Supplement: S6 Fig — A). Insulin secretion following step increase in [Ca2+] at t = 5 min, showing biphasic response. B). Insulin secretion following step increase in glucose at t = 5 min, showing biphasic response. (PDF) [file pcbi.1005116.s010.pdf]

**A**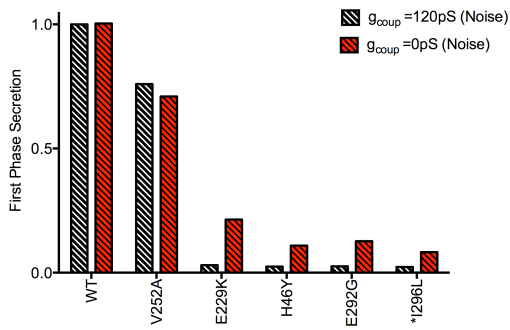**B**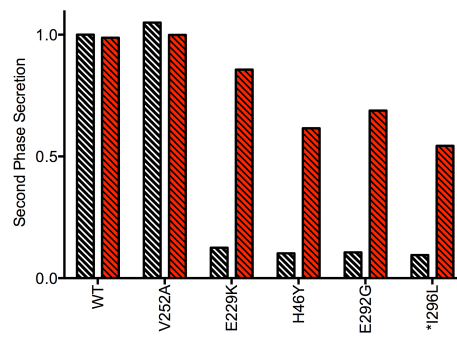**C**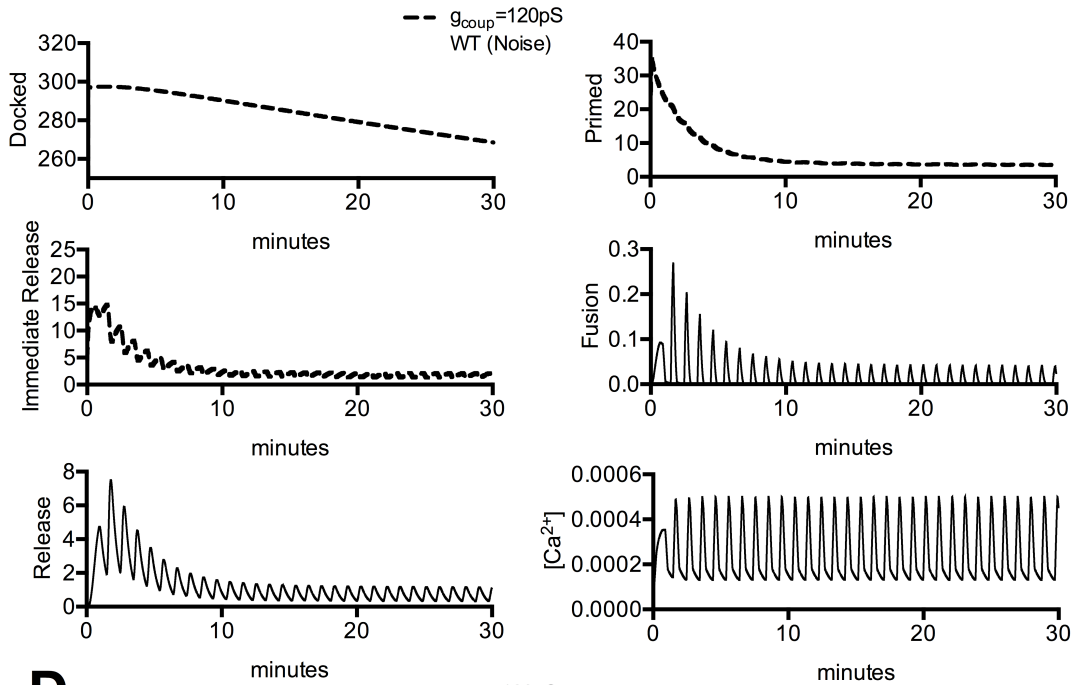**D**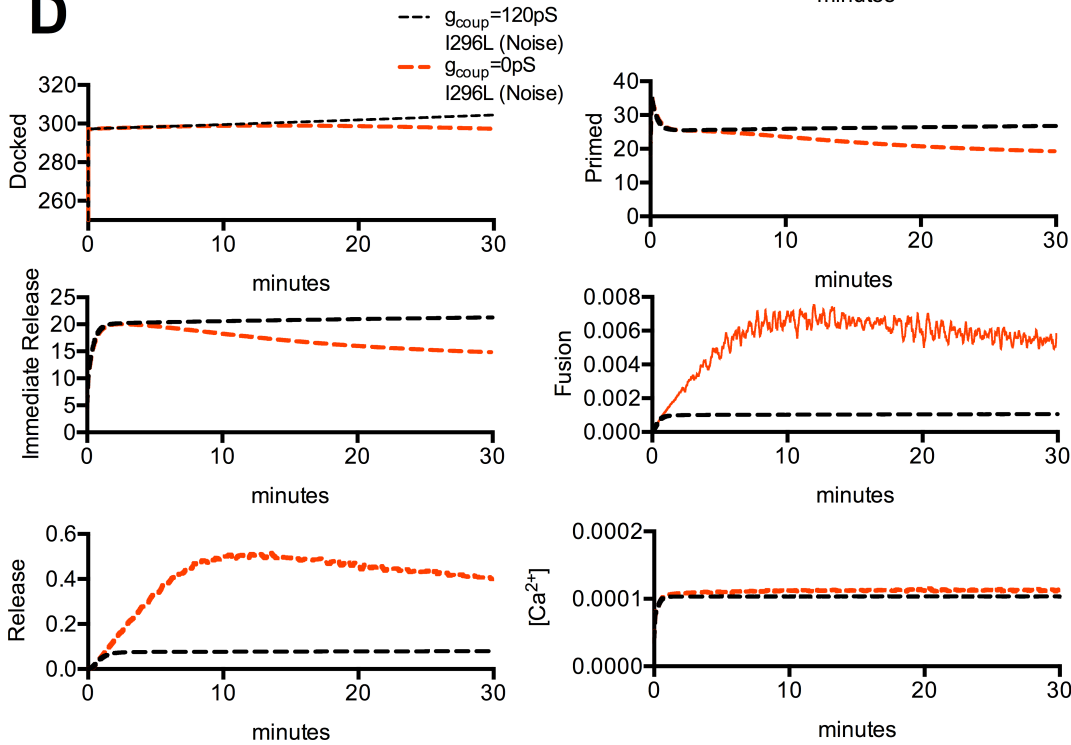

Supplement: S7 Fig — A). Time-averaged insulin secretion over first phase of secretion (t = 0-5min.) for simulations that include mutant KATP channel activity for gcoup = 120pS and gcoup = 0pS, with stochastic channel noise. Simulations include the characterized mutations indicated where reported k’1/2 changes and reported α (if any) are accounted for. B). Time-averaged insulin secretion over second phase of secretion (t = 5-30min.) for simulation conditions in A. C). Time-course of the population of insulin granule pools averaged over a representative simulated WT islet with gcoup = 120pS and stochastic noise included, corresponding to data presented in Fig 8D. D). Time-course of the population of different insulin granule pools, as in C, for a simulated islet that includes I296L mutation for gcoup = 120pS (black) and gcoup = 0pS (red), corresponding to data presented in Fig 8D. All simulations were run at 11mM glucose, Pmut = 1. Data in A,B is presented as mean for n = 2 simulations. (PDF) [file pcbi.1005116.s011.pdf]

$k'_{1/2}$  Reported,  $\alpha$  Estimated (Noise)  
Sulfonylurea Treatment ( $p'_o$ )

**A**

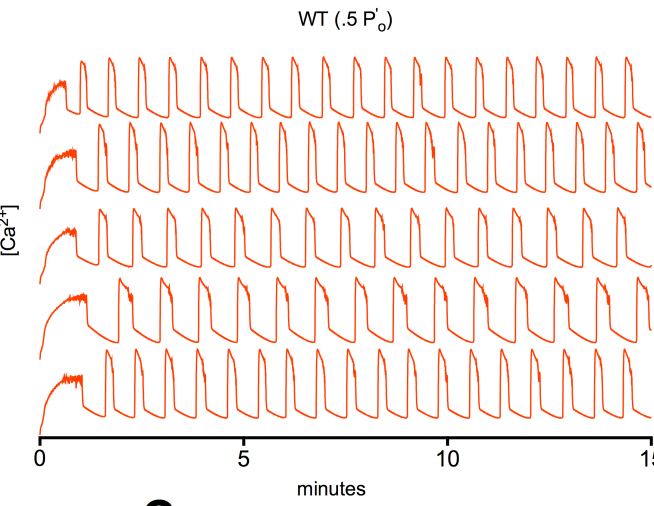

**B**

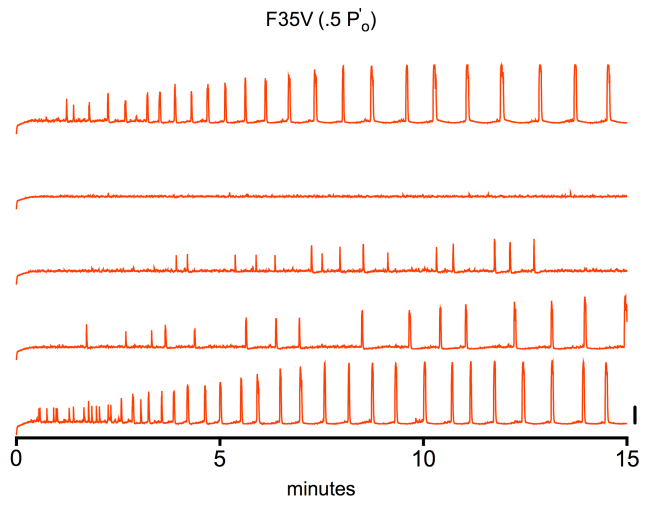

**C**

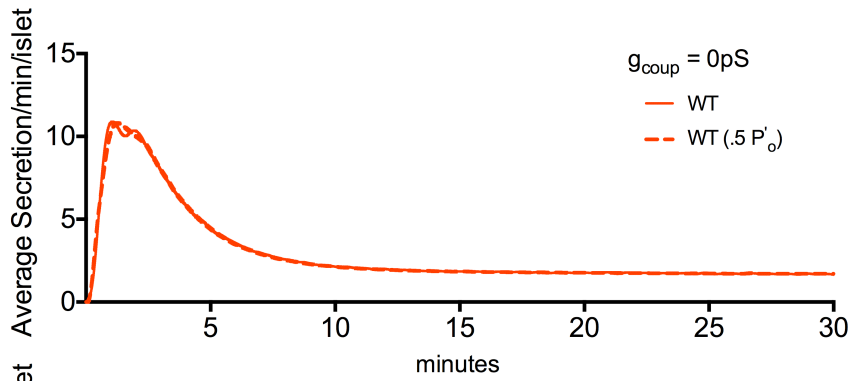

**D**

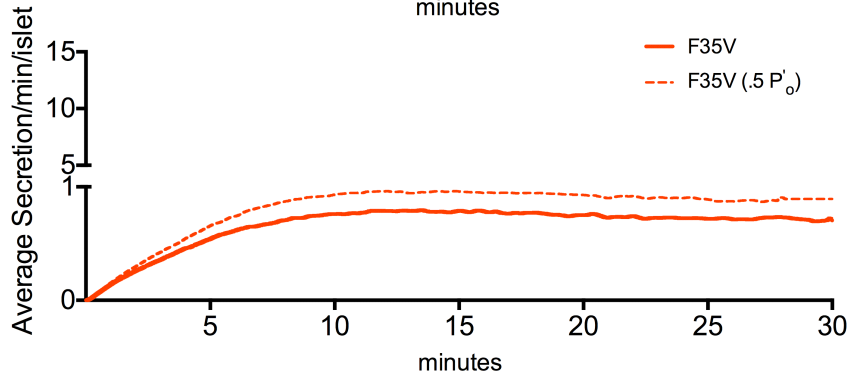

Figure S8

Supplement: S8 Fig — A). Representative [Ca2+] time courses for simulations upon reduced p’o for gcoup = 0pS with stochastic channel noise. B). As in A for simulations that include mutant KATP channel activity. C). Representative time-courses of insulin secretion averaged across the simulated islet for simulations in A, with normal and reduced p’o. D). As in C for simulations in B, with normal and reduced p’o. All simulations were run at 11mM glucose, Pmut = 1. (PDF) [file pcbi.1005116.s012.pdf]

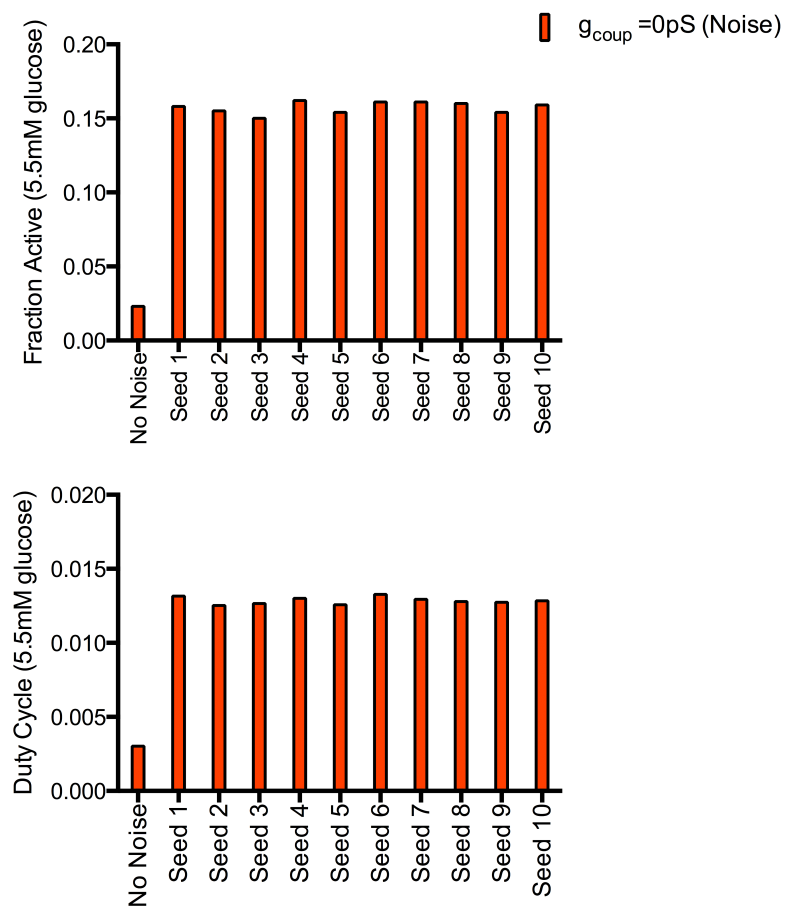

Figure S9

Supplement: S9 Fig — A). Fraction of cells showing significant [Ca2+] elevations for simulations at 5.5mM glucose with gcoup = 0pS, without noise and for 10 different simulations with stochastic channel noise. B). [Ca2+] duty cycle for simulations in A. (PDF) [file pcbi.1005116.s013.pdf]
